# Supplementary material for: Sustained interfacial powering through self-generated mantle and siphon of a gelling droplet
Source: Nat Commun. 2026 Feb 10;17:2566. doi: 10.1038/s41467-026-69481-2 (PMC13000242; doi:10.1038/s41467-026-69481-2)
Supplement: Supplementary file 1 — Supplementary Information [file 41467_2026_69481_MOESM1_ESM.pdf]

Sustained Interfacial Powering through Self-Generated Mantle and Siphon of a Gelling Droplet

Authors: Chunmei Zhou[1,2], Caihong Liu[1,2], Rui Shi[3], Hongxuan Liang[1,2], Hongtu Tan[1,2], Kai Zhuang[1,2], Jiakun Guo[1,2], Xin Tang[1,2]*

Supplementary Notes:

1. Spreading, gelation, and release

Liquid lens:

Upon touching the bath (σ_{ba} = 70 mN m^{-1}), droplet of a surface tension σ_{da} = 42 mN m^{-1} instantaneously spreads into a finite lens with a radius R_l in ~10 ms, during which the surfactant isotropically diffuses at the three-phase contact line. The Marangoni advection lowers the local bath-air surface tension near the boundary, causing the spreading coefficient $S \approx \sigma_{\text{local}} - (\sigma_{\text{da}} - \sigma_{\text{db}})$, where subscripts d, b, a, respectively denote droplet, bath, and air, to be roughly zero.

During spreading, the Reynolds number $\text{Re} \equiv \rho_d u R_l / \mu_d$, where ρ is the mass density, u is the flow velocity, and μ is the dynamic viscosity, is roughly 100 ($\rho_d \approx 10^3$ kg m^{-3}; $u \approx 0.1$ m s^{-1}; $R_l \approx 10^{-3}$ m; $\mu_d \approx 10^{-3}$ Pa s), the viscous effect can be neglect. The capillary-driven spreading is resisted by the inertial force, a scenario similar to the spreading of alcohol droplet on water surface[1,2]. Using the scaling analysis derived by Kim *et al.*, the initial driving force density is $\Delta\sigma / R_s h$ and the inertial effect is $\rho_d R_s / t^2$, where h is the spatio-temporal lens thickness and R_s is the temporal spreading radius. By balancing the two, we can have the time-dependent spreading radius as follows:

$$R_{\mathrm{s}}(t) \sim \left(\frac{\Delta\sigma}{\rho_{\mathrm{d}} h(t)} \right)^{1/2} t \qquad \text{(S1)}$$

To obtain the radius of final liquid lens R_{l} using Equation S1, we need to know the time t_{s} at which the spreading stops. Kim *et al.* assumes that when the surfactant diffusion boundary layer thickness l_{d} equals to the lens thickness h, the spreading stops. Then l_{d} and h are separately derived and made equal to obtain t_{s}. For h, the conservation of mass between initial spherical droplet and final lens disk gives $h \approx V / (\pi R_{\mathrm{s}}^2)$, where V is the droplet volume. For l_{d}, diffusion dynamics provides the thickness of diffusion boundary layer $l_{\mathrm{d}} \approx (D_{\mathrm{s}} t)^{1/2}$, where D_{s} is the surfactant diffusion coefficient which is estimated by the Stokes-Einstein model as $D_{\mathrm{s}} = k_{\mathrm{B}} T / (6\pi\mu_{\mathrm{b}} r)$ with k_{B} the Boltzmann constant, T the temperature, μ_{b} the dynamic viscosity of the bath, and r the hydrodynamic radius of the surfactant. By making $l_{\mathrm{d}} \approx h$, we have $t_{\mathrm{s}} \sim V^2 / \pi^2 R_{\mathrm{s}}^4 D_{\mathrm{s}}$. By substituting t_{s} into Equation S1, we have R_{l} as follows[2]:

$$R_{\mathrm{l}} \sim \left(\frac{\Delta\sigma V^3}{\pi^3 \rho_{\mathrm{d}} D_{\mathrm{s}}^2} \right)^{1/8} \qquad \text{(S2)}$$

The simplification in the assumption of spreading cessation and estimation of diffusion coefficient potentially leaves out a prefactor that is not shown in Equation S2, causing an interception in the logarithmic representation of the measured R_{l} and its scaling prediction. However, the exponent captures the proposed physics for the formation of finite liquid lens.

<u>Gelation and anisotropic release:</u>

For short-timescale perforation, the sodium alginate gelled through chelation with the calcium ions, generating a hydrogel shell (the “mantle”) whose size decreases because of molecule chain aggregation during crosslinking. The shrinking hydrogel carrier of radius $R_{\mathrm{m}}(t)$ increases the curvature $\nabla \cdot \mathbf{n}(t)$ and thus the Laplace pressure of uncrosslinked droplet which

perforates the shell, forming a vent (the "siphon") for surfactant release. For longer-timescale propulsion, the droplet curvature, thus the pressure, is impacted by two factors, that is, the volume of liquid droplet and gradual shrinking $R_m(t)$ caused by continuous diffusion of calcium ions which changes the boundary of droplet. For the flow in siphon, we approximate the liquid discharge as the Poiseuille flow driven by the droplet pressure reflected by the Laplace pressure. By assuming that the siphon has a radius and length of size ~$0.1R_m(t)$, the driving Laplace pressure gradient becomes $\sim \sigma_d \nabla \cdot \mathbf{n}(t) / [0.1 R_m(t)]$ and the viscous resistance is $\sim \mu_d u / 0.01 R_m(t)^2$. By balancing the two, we have $u \sim 0.1 R_m(t) \sigma_d \nabla \cdot \mathbf{n}(t) / \mu_d$. By multiplying the siphon area, we have the liquid volumetric releasing rate as follows:

$$Q \sim 10^{-3} \frac{R_m(t)^3 \sigma_d \nabla \cdot \mathbf{n}(t)}{\mu_d} \qquad \text{(S3)}$$

However, the temporal evolution of curvature for long-timescale is difficult to experimentally capture for the rapid moving motor at millimetre scale. Phenomenologically, releasing rate can be roughly considered as the first-order release suggested by the exponential velocity decay of the motor in Figure 2b. Thus, we can simplify the liquid volumetric releasing rate as follows:

$$Q \sim Q_0 e^{-kt} \qquad \text{(S4)}$$

where Q_0 is the initial volumetric releasing rate $Q_0 = 10^{-3} \frac{R_m(t)^3 \sigma_d \nabla \cdot \mathbf{n}_i}{\mu_d}$ with $\nabla \cdot \mathbf{n}_i$ being droplet-air curvature upon motion initiation, k is the first-order releasing rate constant.

In this way, surfactant releasing rate in mol s^{-1} is as follows:

$$J \sim c_m Q_0 e^{-kt} \qquad \text{(S5)}$$

where c_m is the surfactant concentration in the droplet.

2. Propulsion dynamics

Surfactant transport:

For our isothermal system, the bath surface tension is a function of surfactant surface concentration through a simplified linear equation of state $\sigma = \sigma_0 - \kappa \Gamma$, where $\kappa \equiv -d\sigma / d\Gamma$ and Γ is the surface concentration of surfactant which has a unit of mol m^{-2}. For simplification, we assume that the surfactant is nonvolatile and insoluble so that the evolution of bulk concentration of the surfactant can be neglected. Because the Capillary number is low $\mathrm{Ca} \equiv \mu_b U / \sigma \sim 10^{-2} - 10^{-1}$ ($\mu_b \approx 10^{-3}$ Pa s; $U \approx 0.1$ to 1 m s^{-1}; $\sigma \approx 0.01$ N m^{-1}), liquid bath surface can be considered flat[3]. The surface concentration of surfactant $\Gamma(x, y, t)$ evolves according to the advection-diffusion equation as follows:

$$\frac{\partial \Gamma}{\partial t} + \nabla_s \cdot (\Gamma \mathbf{u}_s) = S_{\mathrm{boundary}} + D_s \nabla_s^2 \Gamma \qquad \text{(S6)}$$

where ∇_s is the surface gradient operator, $\mathbf{u}_s$ is the surface velocity, ∇_s^2 is the Laplacian, the implicit S_{boundary} is the boundary source. We have $D_s \sim 10^{-10}$ m^2 s^{-1}, providing a Péclet number $\mathrm{Pe} \equiv R_m U / D_s \sim 10^6$ ($U \approx 0.1$ m s^{-1}; $R_m \approx 10^{-3}$ m), a transport where advection dominates and thus the diffusion term $D_s \nabla_s^2 \Gamma$ can be neglected.

We assume that in a small but finite time Δt, surfactants are deposited on the liquid surface region swept by the siphon translating at a velocity U without transport (Supplementary Figure 7b). Using such simplified assumption, the amount of released surfactant is $J\Delta t$ and the surface area swept by the siphon is $0.1R_m U \Delta t$. In this way, we have surface concentration of surfactant as follows:

$$\Gamma = J / (0.1 R_m U) \qquad \text{(S7)}$$

This is an over-simplified assumption, especially for regions far behind the motor

(Supplementary Figure 7a,c). In terms of Marangoni propulsion, the surfactant concentration in the vicinity of contact line matters as Marangoni force is the line integral of surface tension acting on motor's perimeter. As shown in Supplementary Figure 7d, near the contact line, such assumption roughly captures the sharp concentration distribution as the time for just released surfactant to transport is limited near the perimeter.

<u>Marangoni propulsion and estimation of surfactant release:</u>

The Marangoni force caused by the local bath surface tension nonequilibrium can be written as follows:

$$\mathbf{F}_{\mathrm{m}} = \int_C \sigma \mathbf{s} dl \qquad \text{(S8)}$$

where $\mathbf{F}_{\mathrm{m}}$ is the Marangoni force, C is the three-phase contact line which is the perimeter of the motor, $\mathbf{s}$ is the unit vector tangent to the free surface and normal to contact line, and l is the arc length along C. Using the simplified assumption of the surfactant distribution, the magnitude of Marangoni force can be written as $F_{\mathrm{m}} \sim \kappa \Gamma \cdot 0.1 R_{\mathrm{m}} = \kappa J / U$.

The Reynolds number during the propulsion is $\mathrm{Re} \sim 10^2 - 10^3$ ($\rho_{\mathrm{b}} \approx 10^3$ kg m^{-3}; $U \approx 0.1$ to 1 m s^{-1}; $R_{\mathrm{m}} \approx 10^{-3}$ m; $\mu_{\mathrm{b}} \approx 10^{-3}$ Pa s). The inertial effect dominates the drag which is associated with the pressure distribution on the motor boundary as $F_{\mathrm{d}} = \rho_{\mathrm{b}} U^2 C_{\mathrm{d}} \pi R_{\mathrm{m}} h_{\mathrm{m}}$, where C_{d} is the drag coefficient and h_{m} is the height of the motor. By balancing F_{m} and F_{d}, we have U as follows:

$$U \sim \sqrt[3]{\frac{\kappa J}{\rho_{\mathrm{b}} C_{\mathrm{d}} \pi R_{\mathrm{m}} h_{\mathrm{m}}}} \qquad \text{(S9)}$$

Substitution of Equation S5 into S9 gives the temporal evolution of propulsion velocity associated with the first-order release of surfactant as $U(t) \sim \left(\frac{\kappa c_{\mathrm{m}} Q_0}{\rho_{\mathrm{b}} C_{\mathrm{d}} \pi R_{\mathrm{m}} h_{\mathrm{m}}} \right)^{1/3} e^{-kt/3}$ where the

coefficient $\left(\frac{\kappa c_{\mathrm{m}} Q_0}{\rho_{\mathrm{b}} C_{\mathrm{d}} \pi R_{\mathrm{m}} h_{\mathrm{m}}}\right)^{1/3}$ with a unit of m s^{-1} is the maximum velocity $U_{\max}$. By referring to the fitted exponential decay of velocity in Figure 2b, we estimate the releasing rate constant to be k~0.0048 s^{-1}, giving a characteristic decay time $1/k$~208 s and $U_{\max}$ = 53 BL s^{-1}.

3. Numerical study of surfactant distribution and motor propulsion

Fundamental equations:

Diluted surfactant transport:

$$\frac{\partial \Gamma}{\partial t} - D_{\mathrm{s}} \nabla_{\mathrm{s}}^2 \Gamma + \mathbf{u}_{\mathrm{s}} \cdot \nabla_{\mathrm{s}} \Gamma = 0 \tag{S10}$$

Note that the boundary source S_{boundary} is set as boundary condition. As a result, it is not shown in the transport equation.

Fluid flow:

$$\rho \frac{\partial \mathbf{u}}{\partial t} + \rho \mathbf{u} \cdot \nabla \mathbf{u} = -\nabla p + \nabla \cdot (\mu \nabla \mathbf{u}) + \mathbf{F}_{\mathrm{st}} + \rho \mathbf{g} \tag{S11}$$

$$\nabla \cdot \mathbf{u} = 0 \tag{S12}$$

where p is pressure, $\mathbf{F}_{\mathrm{st}}$ is surface-tension term including Laplace pressure and Marangoni stress and is calculated using phase field method through Equation S16, $\mathbf{g}$ is gravitational acceleration.

The water-air interface is captured using the phase field method:

$$\frac{\partial \phi}{\partial t} + \mathbf{u} \cdot \nabla \phi = \nabla \cdot \frac{\gamma \lambda}{\varepsilon_{\mathrm{pf}}^2} \nabla \psi \tag{S13}$$

$$\psi = -\nabla \cdot \varepsilon_{\mathrm{pf}}^2 \nabla \phi + \left(\phi^2 - 1\right)\phi + \frac{\varepsilon_{\mathrm{pf}}^2}{\lambda}\left(\partial f / \partial \phi\right) \tag{S14}$$

$$\lambda = \frac{3\varepsilon_{\mathrm{pf}}\sigma}{\sqrt{8}}, \quad \gamma = \chi \varepsilon_{\mathrm{pf}}^2 \tag{S15}$$

Two phase flow:

$$\mathbf{F}_{\text{st}} = \left(\frac{\lambda}{\varepsilon_{\text{pf}}^2}\psi - \frac{\partial f}{\partial \phi}\right)\nabla\phi + \left(\frac{|\nabla\phi|^2}{2} + \frac{(\phi^2-1)^2}{4\varepsilon_{\text{pf}}^2}\right)\nabla\lambda - (\nabla\lambda\cdot\nabla\phi)\nabla\phi \quad \text{(S16)}$$

$$\rho = \rho_1 V_{\text{f},1} + \rho_2 V_{\text{f},2} \quad \text{(S17)}$$

$$\mu = \mu_1 V_{\text{f},1} + \mu_2 V_{\text{f},2} \quad \text{(S18)}$$

$$V_{\text{f},1} = \frac{1-\phi}{2},\ V_{\text{f},2} = \frac{1+\phi}{2},\ V_{\text{f},1} + V_{\text{f},2} = 1 \quad \text{(S19)}$$

where ϕ is phase field variable, ε_{pf} is the parameter controlling interface thickness, Ψ is phase field help variable, $\partial f/\partial\phi$ is the ϕ-derivative of external free energy which is 0 in our model, χ is the mobility tuning parameter, **n** is the surface outward unit normal vector,ρ_1 and ρ_2 represent fluid density, μ_1 and μ_2 represent dynamic viscosity, $V_{\text{f},1}$ and $V_{\text{f},2}$ represent volume fraction of fluid 1 and 2. The term $\left(\frac{|\nabla\phi|^2}{2} + \frac{(\phi^2-1)^2}{4\varepsilon_{\text{pf}}^2}\right)\nabla\lambda - (\nabla\lambda\cdot\nabla\phi)\nabla\phi$ represents the introduced surface tension gradient effect. σ is surface tension, which is a function of surfactant concentration Γ.

Motion and deformation of motor (linear elastic material with negligible deformation):

$$\rho\frac{\partial^2 \mathbf{u}_{\text{solid}}}{\partial t^2} = \nabla\cdot([C]:[S]) + \rho\mathbf{g} \quad \text{(S20)}$$

where $\mathbf{u}_{\text{solid}}$ is displacement, $[C]$ is elasticity tensor, $[S]$ is strain tensor. Note that for computation efficiency, motor's motion in z axis is neglected, thus force acting on the solid in z axis such as gravitational one ρ**g** is set to be 0. Because of negligible deformation, governing equation for motion of motor can be written in integral form as follows:

$$m\mathbf{a} = \int_C \sigma\mathbf{s}dl + \int_{Ac}(-p\mathbf{I} + \mu\nabla\mathbf{u})\cdot\mathbf{n}dA \quad \text{(S21)}$$

where m is motor's mass, **a** is motor's acceleration, C is the three-phase contact line, **s** is the

unit vector tangent to the free surface and normal to contact line, l is the arc length along C, $\mathbf{I}$ is the identity matrix, Ac is the area of motor, A is the differential area.

Boundary conditions:

Liquid-solid interface:

All solid boundaries are considered as no slip boundaries and wetted wall with a specified contact angle of 90 °.

Surfactant release:

For surfactant release, a Neumann condition of prescribed flux (1.5 mol m^{-2} s^{-1}) is set on a part of motor's side area of an arc length of $0.1R_m$ (a segment of motor's perimeter with a length of $0.1R_m$) and height of h_m. Zero flux condition is set at the other part of motor's surface. The concentration-dependent surface tension is applied as boundary load on the motor.

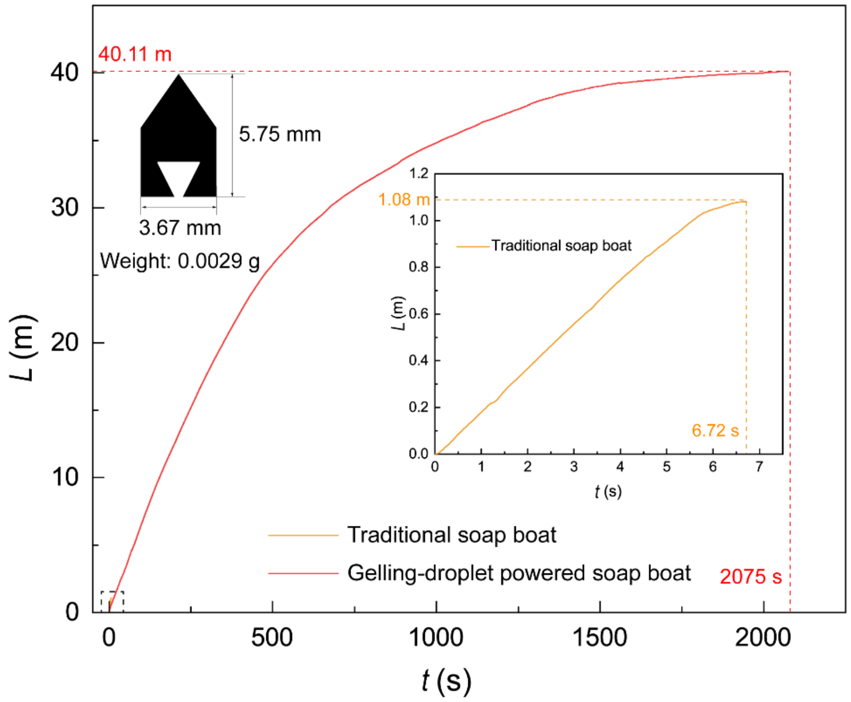

Supplementary Figure 1. Lifetime comparison. Comparison of propulsion performance of a conventional soap boat powered by traditional fuel dripping and the gelling-droplet motor (artificial mantle-siphon strategy). The propulsion is shown in Supplementary Movie 1. In both cases, same amount of surfactant is used (0.004 g PEGDA400). Top left inset shows the geometry of the boat. In both cases, boats are fabricated from a polyvinyl butyral sheet and has the same weight of 0.0029 g. The gelling-droplet powered one has extra droplet weight of 0.016 g. For the gelling-droplet powered one, it has the maximum velocity of 141.99 mm s^{-1} and an average velocity of 19.33 mm s^{-1}.

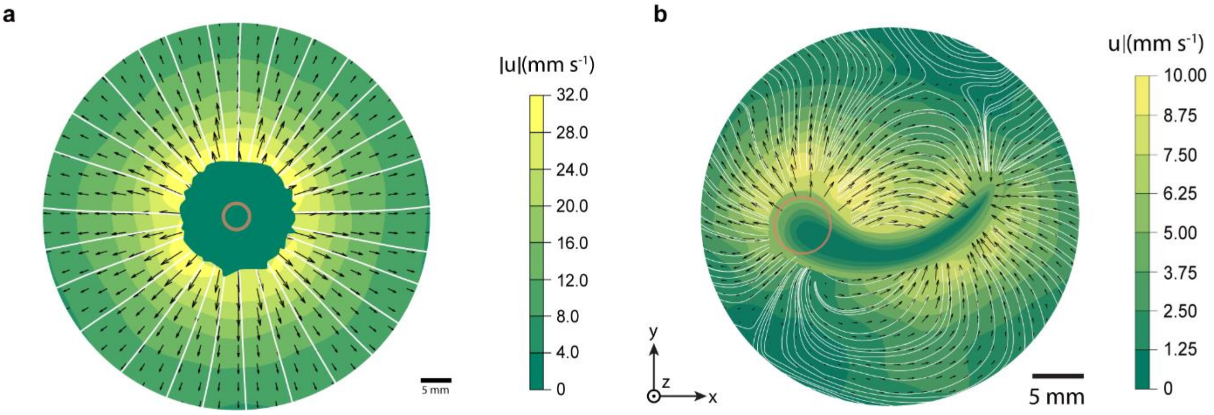

Supplementary Figure 2. Top-view PIV mapping. (a) Isotropic release during spreading. The nearly circular zero-velocity region in the top-view PIV measurements signals the isotropic surfactant release upon droplet spreading. The red circle denotes the motor. (b) Anisotropic release during propulsion. The Marangoni stress sweeps away tracer particles, forming regions appeared to have zero velocities. Upon propulsion, the surfactant release concentrates at the motor rear. The red circle denotes the motor (Supplementary Movie 4).

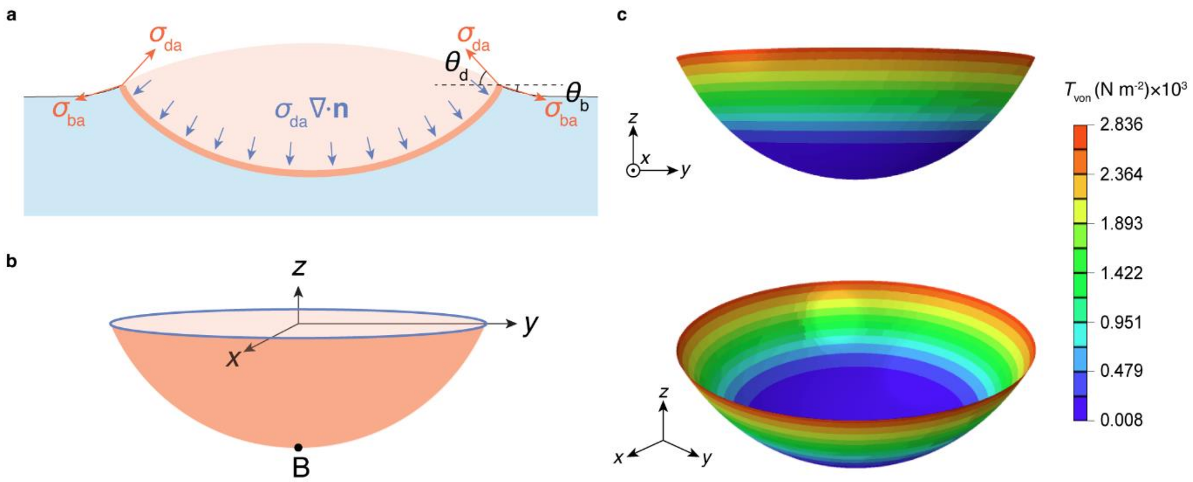

Supplementary Figure 3. Stress in mantle before perforation. (a) Because of negligible hydrostatic force, the mantle is subjected to surface tension force σ_{da}, σ_{ba} and Laplace pressure $\sigma_{da}\nabla\cdot\mathbf{n}$. (b) Schematics of configuration for finite element analysis. Surface tension forces, σ_{da} = 42 mN m^{-1} and σ_{ba} = 70 mN m^{-1}, act along the contact line which is denoted blue. Laplace pressure, $\sigma_{da}\nabla\cdot\mathbf{n}$ = 12.24 N m^{-2}, acts on inner surface of the mantle. Based on experiment, θ_d = 30˚ and θ_b = 2˚. Centre of contact line ring is set as origin. B(0, 0, -1 mm) is the bottom point of the mantle. R_m is set to be 2.8 mm. For boundary conditions, the contact line remains on water surface, thus its displacement in z-direction is 0. Because of axisymmetric configuration, the displacement of point B in x- and y-direction is 0. Properties of the calcium alginate gel are set as follows: Young's modulus is 30 kPa, Poisson's ratio is 0.5, and density is 2200 kg m^{-3}. (c) The numerically-calculated von Mises stress (T_{von}) in the mantle. The maximum stress appears at the perimeter where perforation preferentially occurs.

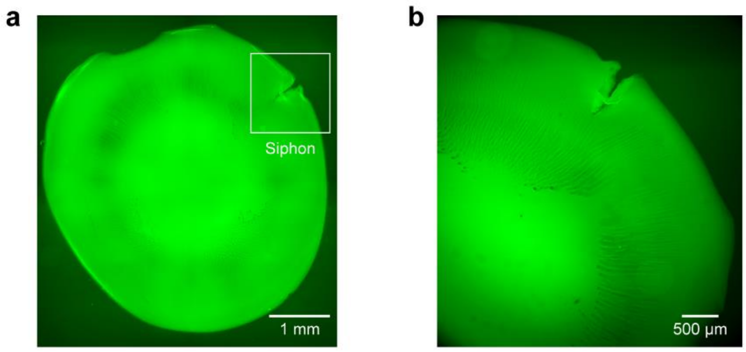

Supplementary Figure 4. Siphon formed by gel fracture. Fluorescence image of motor (a) and magnified siphon (b) showing that the releasing hole appears as a fracture on the gel mantle. The opening has a maximum width of roughly 6% of motor's radius.

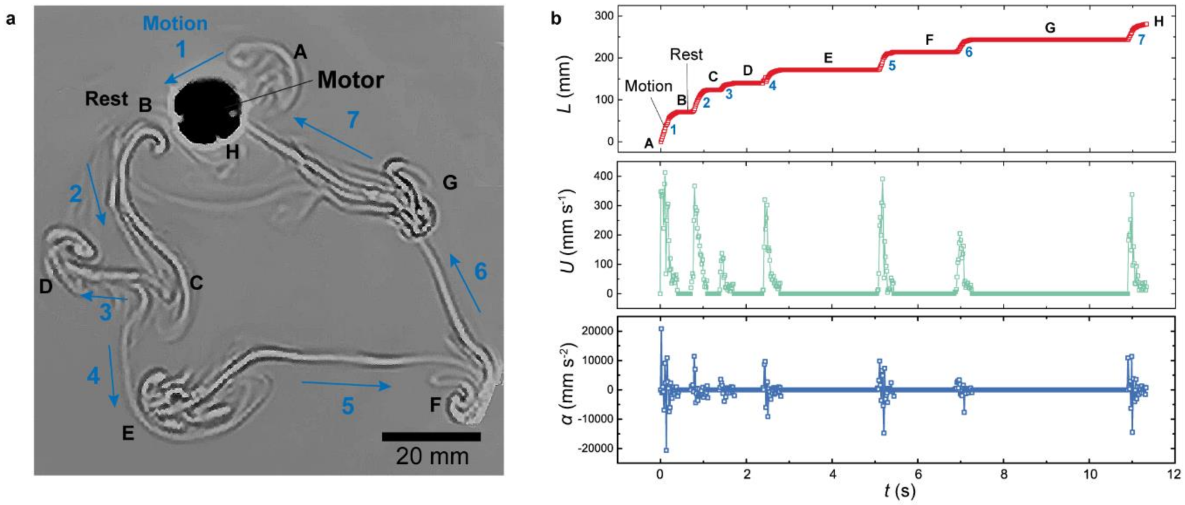

Supplementary Figure 5. Pulsed motion. (a) Image of a motor after 7 pulsed motions (Supplementary Movie 5). (b) Displacement, velocity, and acceleration of the 7 pulsed motions.

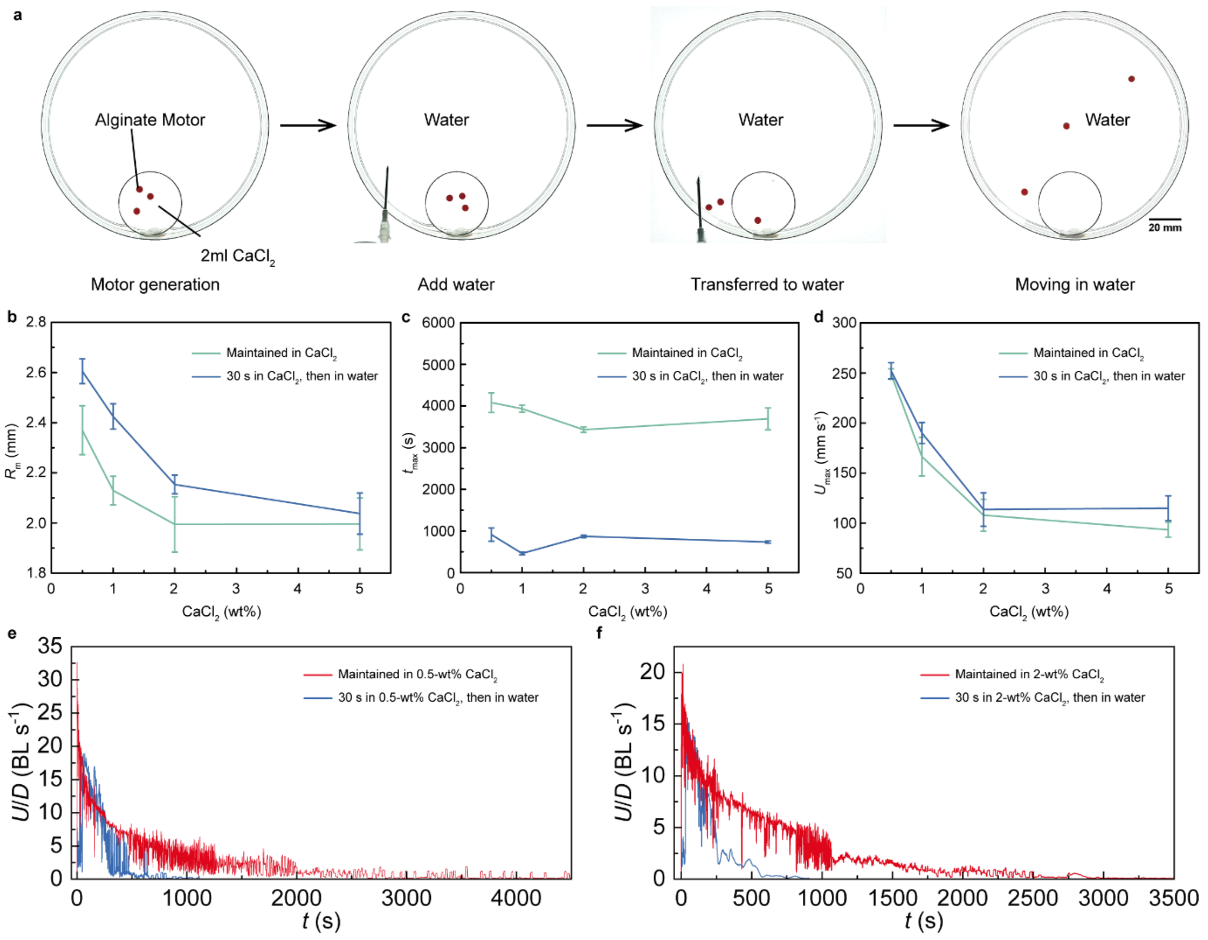

Supplementary Figure 6. Continuous crosslinking during self-propulsion. (a) Micromotors are formed in $CaCl_2$ liquid bath for ~30 s and then transferred into water whereupon crosslinking is largely terminated (see Supplementary Movie 6). Comparison of micromotor's radius R_m (b), lifetime t_{max} (c), and maximum velocity U_{max} (d) for ones maintained in $CaCl_2$ (crosslinking persists for the entire lifetime) and ones transferred into water after ~30 s (crosslinking only persists for the first 30 s). Note that R_m in (b) is measured at the end of micromotor's lifetime. Error bars denote standard deviation of 3 experiments. The normalized propelling velocity as a function of time for 0.5-wt% (e) and 2-wt% (f) $CaCl_2$ solution.

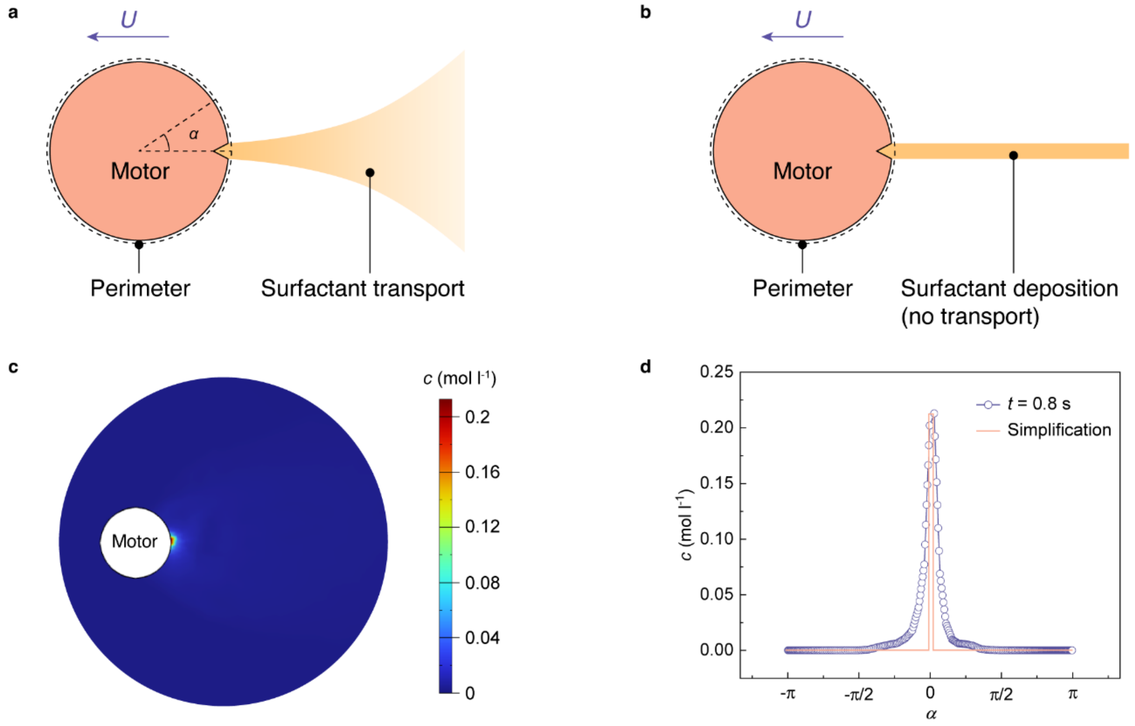

Supplementary Figure 7. Evolution of surfactant distribution. Schematics of concentration distribution with (a) and without (b) surfactant transport through diffusion and advection. (c) Numerically-calculated distribution of surfactant at t = 0.8 s (Supplementary Movie 7). (d) Numerically-calculated surfactant concentration along the perimeter of motor. The surfactant is concentrated around the releasing siphon. In derivation of Equation S7, we simplify the concentration around the perimeter as a pulse function denoted by the red line.

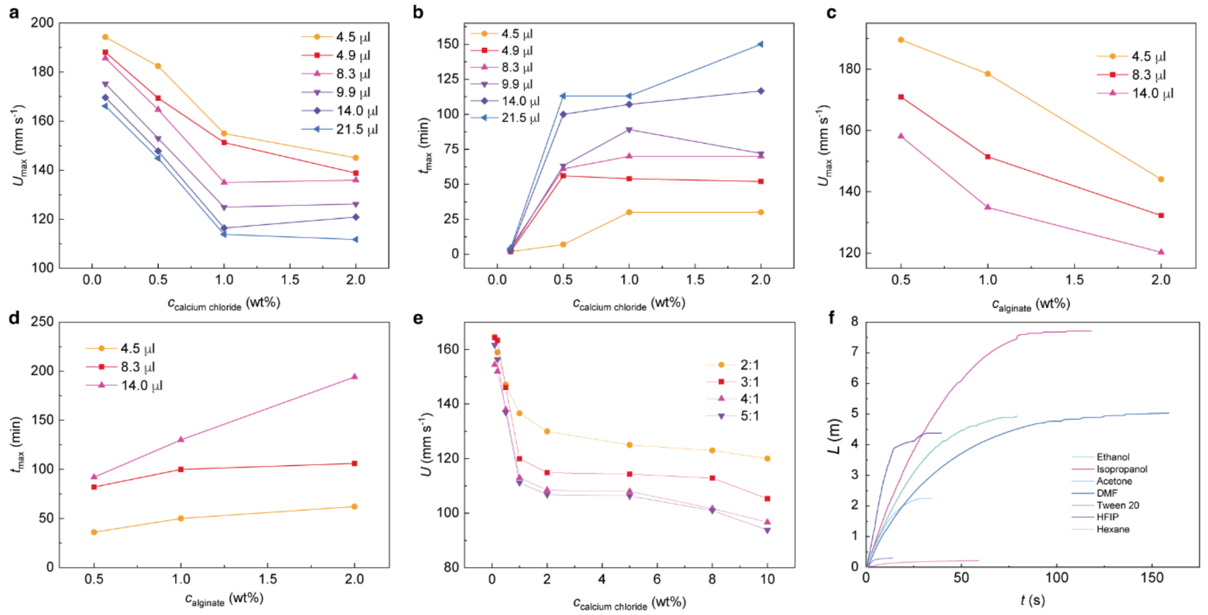

Supplementary Figure 8. Impact of crosslinking. As the crosslinking strengthens, the maximum propulsion velocity U_{max} decreases and the motor lifetime t_{max} increases. As the concentration of alginate or calcium chloride increases, U_{max} decreases (a,c) and t_{max} increases (b,d). (e) Average propulsion velocities U as a function of concentration of calcium chloride. Colour denotes mass ratio of sodium alginate to PEGDA. (f) The temporal positions of motors driven by small molecule surfactants.

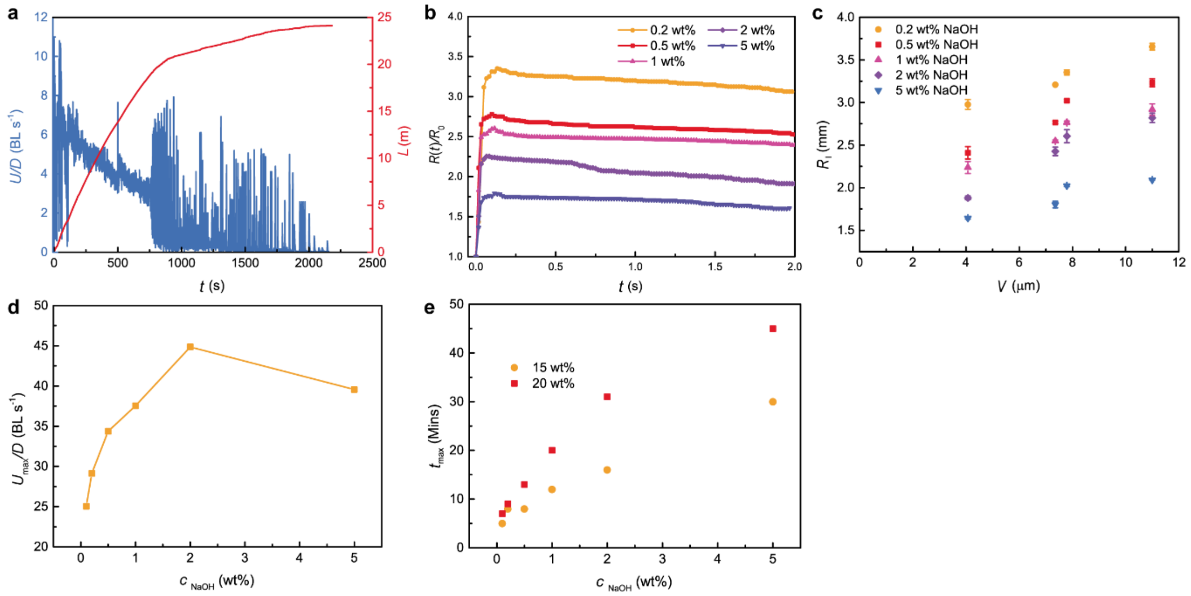

Supplementary Figure 9. Chitosan motor. (a) Displacement and propelling velocity normalized by motor diameter D as a function of time (precursor: aqueous solution containing 5-wt% chitosan and 20-wt% PEGDA400; bath: 10-wt% NaOH aqueous solution). (b) Temporal evolution of motor radius $R(t)/R_0$, with t = 0 defined as the moment when the precursor droplet contacts the bath. (c) Measured finite spreading radius R_l of the chitosan motor as a function of precursor droplet volume V. Error bars denote standard deviation of 3 experiments. (d) Effect of NaOH concentration on maximum velocity normalized by motor diameter D. (e) Effect of NaOH concentration on lifetime t_{max}.

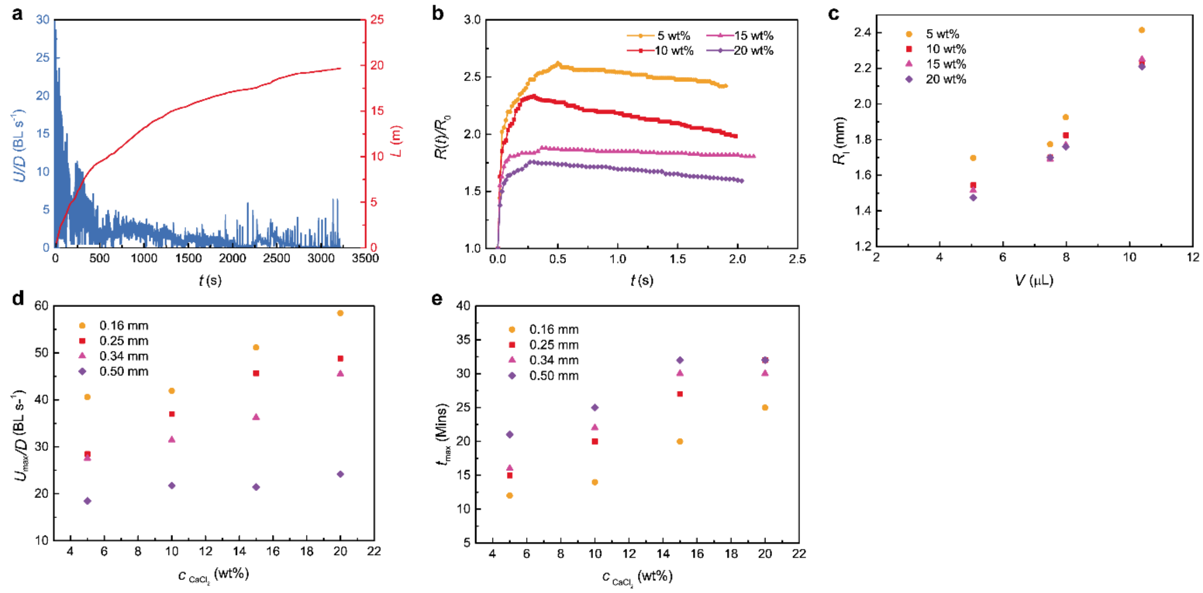

Supplementary Figure 10. Carboxymethyl cellulose calcium (CMC Ca) motor. (a) Displacement and propelling velocity normalized by motor diameter D as a function of time (precursor: aqueous solution containing 1.5-wt% CMC and 25-wt% PEGDA400; bath: 1 wt% $CaCl_2$ aqueous solution). (b) Temporal evolution of CMC motor radius $R(\mathrm{t})/R_0$, with t = 0 defined as the moment when the precursor droplet touches the bath. (c) Measured finite spreading radius R_l of the CMC motor as a function of precursor droplet volume V. (d) Effect of $CaCl_2$ concentration on maximum velocity normalized by motor diameter D. (e) Effect of $CaCl_2$ concentration on motor lifetime t_{max}.

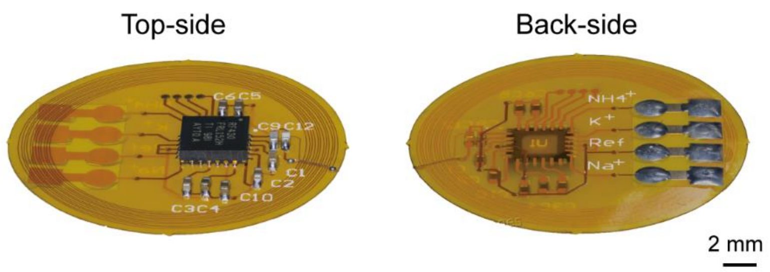

Supplementary Figure 11. Powering the sensing system. Photographs of the sensing system without encapsulation. Left: top view; right: back view.

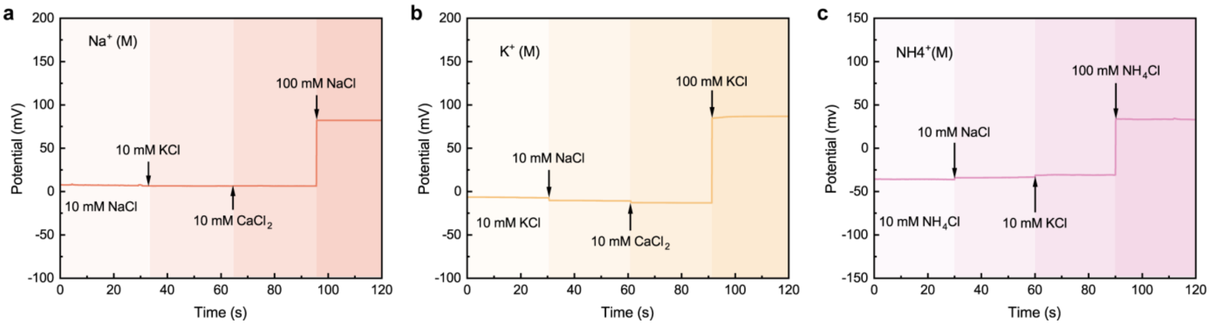

Supplementary Figure 12. Selectivity of ion sensor. (a) Anti-interference characterization of the Na^+ sensor. The Na^+ sensor shows no response to K^+ and Ca^{2+} ions. (b) Anti-interference characterization of the K^+ sensor. The K^+ sensor shows no response to Na^+ and Ca^{2+} ions. (c) Anti-interference characterization of the NH_4^+ sensor. The NH_4^+ sensor shows no response to Na^+ and K^+ ions.

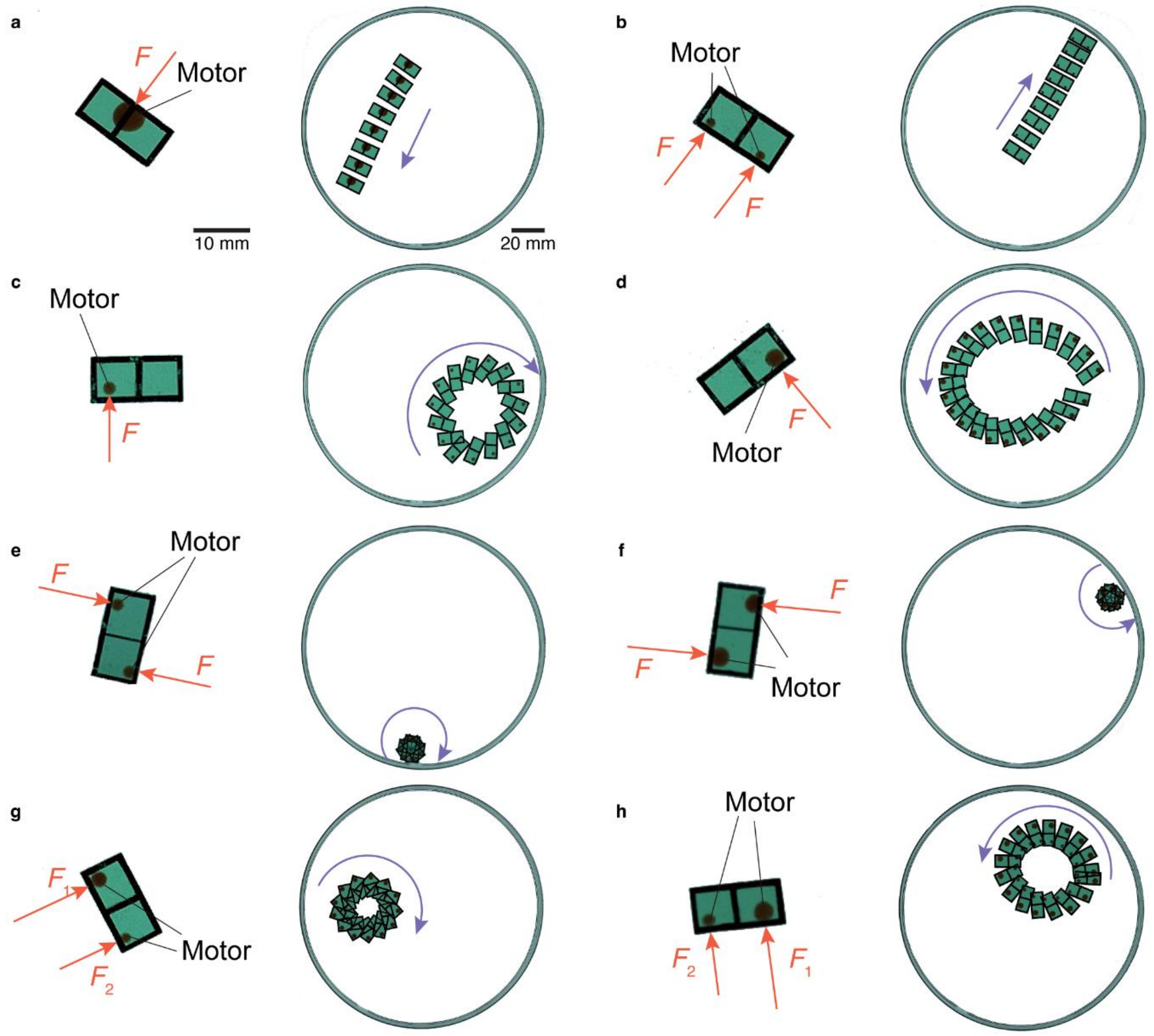

Supplementary Figure 13. Control of free interfacial machines. (a, b) Symmetric motor dispensing drives the machine into linear translation. (c, d, g, h) Asymmetric motor dispensing drives the machine into curvilinear translation. (e, f) diagonal motor dispensing drives the machine into rotation (Supplementary Movie 10).

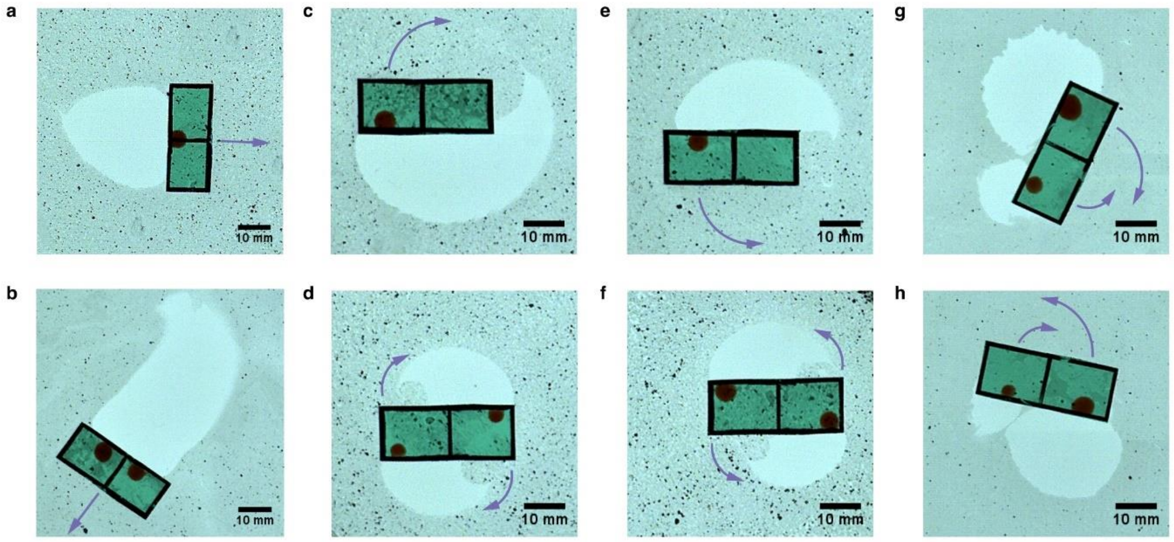

Supplementary Figure 14. Surfactant release of interfacial machines. Surfactant release of motors drive machines into linear translation (a, b), curvilinear translation (c, e, g, h), and rotation (d, f). The area of tracer-free region qualitatively correlates with the magnitude of the propulsion force (Supplementary Movie 10).

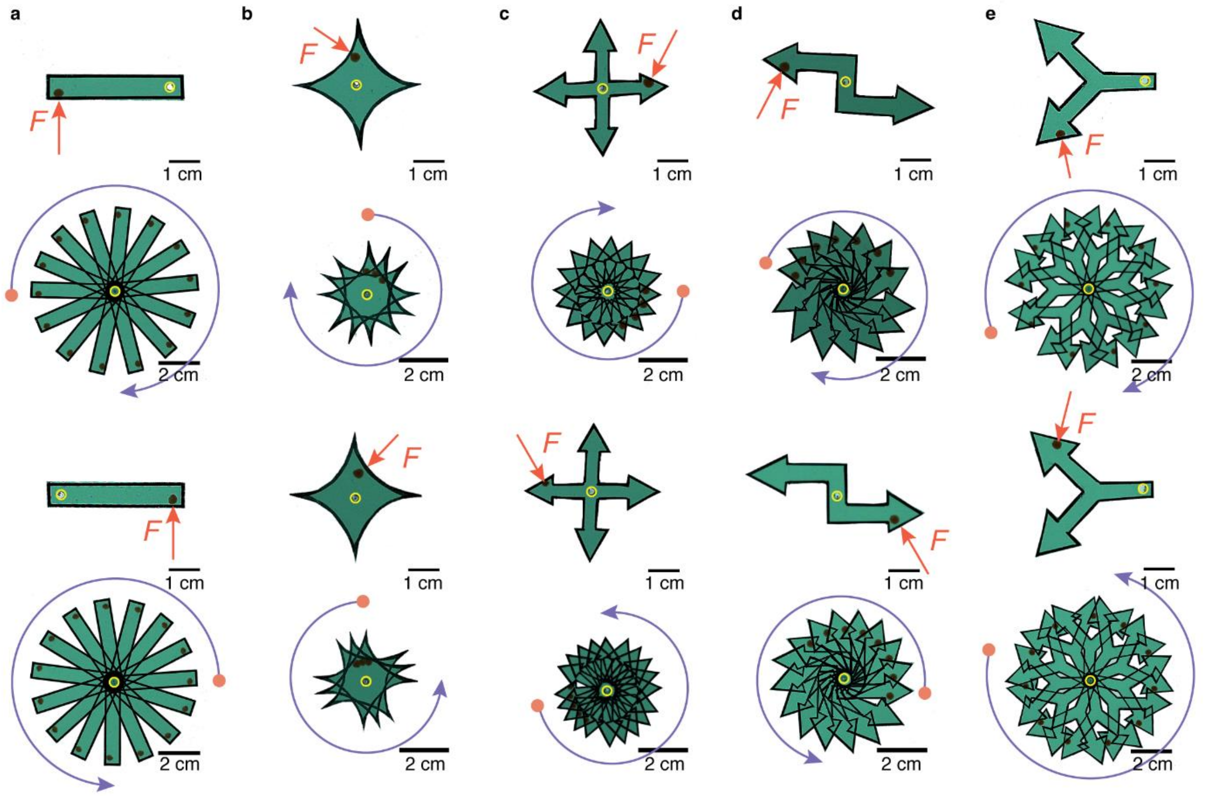

Supplementary Figure 15. Control of pinned interfacial machines. The rotation direction of the bar (a), four-tooth gear (b), four-tip arrow (c), two-tip arrow (d), and branched arrow (e) can be controlled by the offset motor dispensing. Yellow circles denote fixed axles (Supplementary Movie 11).

Supplementary Table 1. List of symbols.

R_s	Radius of spreading droplet	m
R_l	Radius of finite liquid lens	m
h	Thickness of liquid lens	m
h_m	Height of motor	m
R_m	Radius of partially crosslinked motor	m
r	Hydrodynamic radius of surfactant	m
D	Diameter of crosslinked motor	m
B	Body length	m
L	Propulsion distance	m
l_d	Thickness of the diffusion boundary layer	m
C	Length of three-phase contact line	m
l	Arc length along C	m
V	Volume	m^3
U	Spreading or propulsion velocity	$m\ s^{-1}$
$\mathbf{a}$	Acceleration	$m\ s^{-2}$
$\mathbf{u}_s$	Surface flow velocity	$m\ s^{-1}$
$\mathbf{u}$	Bulk flow velocity	$m\ s^{-1}$
u	Magnitude of bulk flow velocity	$m\ s^{-1}$
$\mathbf{F}$	Force	N
F	Magnitude of force	N
p	Pressure	Pa
$\mathbf{g}$	Gravitational acceleration	$m\ s^{-2}$
ρ	Mass density	$kg\ m^{-3}$
Γ	Surface concentration of surfactant	$mol\ m^{-2}$
$S_{boundary}$	Boundary source	$mol\ m^{-2}\ s^{-1}$
c	Concentration	$mol\ m^{-3}$
c_m	Surfactant concentration in the droplet	$mol\ m^{-3}$
J	Surfactant release rate	$mol\ s^{-1}$
Q	Liquid volumetric releasing rate	$m^3\ s^{-1}$
Q_0	Initial volumetric releasing rate	$m^3\ s^{-1}$
k	First-order releasing rate constant	s^{-1}
κ	$\kappa \equiv -d\sigma/d\Gamma$	$N\ mol^{-1}\ m$

D_s	Surface diffusivity of surfactant	$m^2\ s^{-1}$
k_B	Boltzmann constant	$J\ K^{-1}$
T	Temperature	K
V_e	Electric potential	V
μ	Dynamic viscosity	Pa s
σ	Surface or interfacial tension	$N\ m^{-1}$
S	Spreading coefficient	$N\ m^{-1}$
t	Time	s
t_s	Spreading time	s
t_{max}	Lifetime of motor	s
Re	Reynolds number	/
Pe	Péclet number	/
Ca	Capillary number	/
C_d	Coefficient of drag	/
**s**	Unit vector tangent to the free surface and normal to the contact line	/
**n**	Surface unit normal vector	/

1

Supplementary Table 2. Properties of precursor and bath solutions.

Sample		Density (g ml^{-1})	Surface tension (mN m^{-1})	Sample		Density (g ml^{-1})	Surface tension (mN m^{-1})
Alginate solution	0.5 wt% Sodium alginate (SA)	0.983	64.94	Motor precursor solution	0.75 wt% SA, 25 wt% PEGDA-400	1.0533	42.53
	1 wt% SA	1.001	64.21		0.75 wt% SA, 25 wt% PEG-300	1.061	54.68
	2 wt% SA	1.004	59.29		0.75 wt% SA, 25 wt% PEG-400	1.058	55.41
	3 wt% SA	1.007	53.45		0.75 wt% SA, 25 wt% Ethanol	0.969	33.51
Bath solution	0.1 wt% $CaCl_2$	0.9831	69.84		0.75 wt% SA, 25 wt% Isopropanol	0.964	28.65
	0.2 wt% $CaCl_2$	0.98362	72.00		0.75 wt% SA, 25 wt% Acetone	0.942	38.27
	0.5 wt% $CaCl_2$	0.995	69.71		0.75 wt% SA, 25 wt% Dimethylformamide	0.984	51.18
	1 wt% $CaCl_2$	1.00115	69.99		0.75 wt% SA, 25 wt% Hexane	0.932	18.7
	2 wt% $CaCl_2$	1.00535	71.17		0.75 wt% SA, 25 wt% Hexafluoroisopropanol	1.146	14.7
	5 wt% $CaCl_2$	1.03265	71.24		0.75 wt% SA, 25 wt% Tween 20	1.061	/
	8 wt% $CaCl_2$	1.0585	71.38				
	10 wt% $CaCl_2$	1.0743	73.07				

Supplementary Table 3. Properties of chemical fuels.

Chemical fuel	Molecular weight M (g mol^{-1})	Density ρ (g ml^{-1})	Surface tension σ (mN m^{-1})	Dynamic viscosity μ (mPa s)	HLB	Solubility in water
PEGDA-400	400	1.110	40.81	57	/	√
PEG-300	300	1.103	44.90	73	20	√
PEG-400	400	1.084	46.6	95.22	20	√
Ethanol	46.07	0.789	33.0	1.040	1	√
Isopropanol (IPA)	60.1	0.785	22.3/22.6	2.4	/	√
Acetone	58.08	0.784	23.7	0.36	16.7	√
Dimethylformamide (DMF)	73.095	0.944	37.1	0.92	/	√
Hexane	86.18	0.655	18.4	0.31	/	×
Hexafluoroisopropanol (HFIP)	168.04	1.596	14.7	1.65	/	√
Tween 20	604.42	1.1	/	370-430	16.7	√

Supplementary Table 4. Motors driven by different chemical fuels.

Chemical fuel	Mass m (mg)	Volume V (μl)	Diameter D (mm)	Maximum velocity U_{max} (mm s^{-1})	Relative velocity U_{max}/D (BL s^{-1})	Lifetime t_{max} (s)	Displacement L_{max} (mm)
PEGDA-400	5.90	4.75	3.33±0.055	147.12	44.18	6000	77768.6
PEG-300	6.05	6.97	3.02±0.179	83.465	27.67	~5400	75398
PEG-400	7.86	7.43	3.025±0.061	95.159	31.46	~4500	66221
Ethanol	4.02	4.15	5.227 ±0.380	206.027 ±5.38	39.419	~101 ±6.18	~4930
Isopropanol (IPA)	4.46	4.62	3.954 ±0.148	216.705	54.811	~182 ±44.45	~7719
Acetone	4.1	4.35	5.177 ± 0.663	215.984 ±3.685	41.72	~33.5	~2115 ± 94
Dimethylformamide (DMF)	8.16	8.29	7.195 ±0.277	170.335 ±4.092	23.67	258.7	~5030
Hexane	7.45	7.99	4.406 ± 0.038	30.392	6.898	~55	213.42
Hexafluoroisopropanol (HFIP)	2.965	2.59	4.592 ± 0.388	541.991 ± 14.198	118.026	~70.5	~4379
Tween 20	6.51	6.13	5.734 ± 0.087	154.424 ± 1.382	26.93	~14 s	299.193

Note: These experiments were conducted under the same conditions. Motor precursor solution: 0.75 wt% sodium alginate and 25 wt% chemical fuel; Bath solution: 1 wt% calcium chloride solution.

Supplementary Table 5. Chemical Marangoni motor benchmarking[4].

Motor types	Motor material	Fuels	Legend	Maximum velocity (mm s^{-1})	Motor output α_{max} (mm^{-2} s^{-1})	Motor efficiency ε_{max} (μJ g^{-1})	Ref.
Our work (PEGDA)	Alginate calcium	PEGDA-400	A1	894.28	206000	1600	Present
	Alginate calcium	PEGDA-400	A2	956.29	144000	1830	Present
	Alginate calcium	PEGDA-400	A3	669.35	175000	896	Present
	Alginate calcium	PEGDA-400	A4	517.27	3490	535	Present
	Alginate calcium	PEGDA-400	A5	345.17	1120	238	Present
	Alginate calcium	PEGDA-400	A6	307.68	110	189	Present
	Alginate calcium	PEGDA-400	A7	278.01	17.5	154	Present
	Alginate calcium	PEGDA-400	A8	258.57	5.08	133	Present
	Alginate calcium	PEGDA-400	A9	192.02	0.94	73	Present
Our work (HFIP)	Alginate calcium	HFIP	B1	736.465	422	1080	Present
	Alginate calcium	HFIP	B2	688.908	25.6	949	Present
	Alginate calcium	HFIP	B3	650.12	16.4	845	Present
	Alginate calcium	HFIP	B4	560.953	10.4	629	Present
Protein motor	SRT protein	HFIP	C1	408.2	3800000	417	[4]
	SRT protein	HFIP	C2	385.3	401000	371	[4]
	SRT protein	HFIP	C3	340.6	128000	290	[4]
	SRT protein	HFIP	C4	250.3	23500	157	[4]
	SRT protein	HFIP	C5	216.3	2260	117	[4]
	SRT protein	HFIP	C6	156.9	589	61.6	[4]
	SRT protein	HFIP	C7	148.5	139	55.1	[4]
Droplet	Aniline oil droplet	Aniline oil	D	37.5	0.0764	0.8	[5]
	Pentanol droplet	Pentanol	E	~49	7.95	1.25	[6]
	PVDF droplet	DMF	F	79	1.24	4.46	[7]
Boats	PECA-loaded pipet tip	Ethanol	G	90	0.3	20.3	[8]

	Camphor disk	Camphor	H	60	7.84	1.80	9
	1, 10-Benzoquinone disk	Benzoquinone	I	17.6 ± 4.1	3.39	0.288	10
	Phenanthroline disk	Phenanthroline	J	/	28	0.242	11
	PDMS boat	IPA	K	85	0.043	51.6	12
	SU-8 boat	IPA	L	330	6.04	57.9	13
	SU-8 boat	IPA	M	140	2.12	49	14
Polymer capsules & gels	PNIPAm gel	Ethanol	N	310	73.5	125	15
	Polysulfone capsule	DMF/SDS	O	157	8.96	14	16
	Poly(ADA-co-AA) gel	THF	P	46.8	2	1.39	17
	Agarose gel	Camphor	K	3	50.6	0.499	18
	Cellulose acetate	Peppermint oil	R	150	17.7	0.023	19
	Multi-stage hydrogel rocket	SDS	S	16.2 ± 1.3	0.253	0.772	20
	Polyacrylamide hydrogel	Ethanol	T	2.2	0.25	0.00005	21
MOF swimmers	Peptide-MOF (HKUST-1)	DPA	U	31.8 ± 1.2	45	3.97	22
	Porphyrin(PCN-222) MOF	BA/DMF	V	120	48	22	23
	PCN-222 MOF	DBF	W	210	84	57.7	23
	Peptide-MOF (CuJAST-1)	DPA	X	67	67	3.06	24

Abbreviation: MOF: Metal-Organic Frameworks (MOF); PEGDA: Poly (ethylene glycol) diacrylate (PEGDA); HFIP: Hexafluoroisopropanol (HFIP); PVDF: Polyvinylidene fluoride (PVDF); PNIPAm: Poly-N-isopropylacrylamide (PNIPAm); PECA: poly (2-ethyl cyanoacrylate) (PECA); PDMS: Polydimethylsiloxane (PDMS); DMF: N,N-dimethylformamide (DMF); IPA: Isopropanol; Isopropyl alcohol (IPA); THF: Tetrahydrofuran (THF); SDS: Sodium dodecyl sulphate (SDS); DPA: Diphenylalanine (DPA); BA: Benzoic acid (BA); DBF: N, N-dibutylformamide (DBF).

Supplementary Table 6. Summary of performances for insects, terrestrial and aquatic animals, artificial robots.

Category	Operation environment	Name	Actuation mechanis	Body mass (m, g)	Body length (B, mm)	Maximum velocity (U_{max}, mm s^{-1})	Relative velocity (U/B, BL s-1)	Ref.
Mammals (Terrestrial animals)	Terrestrial	Mouse	/	40	100	3600	35.7	[25]
	Terrestrial	Cat	/	4000	250	13000	53.6	[26]
	Terrestrial	Dog	/	30000	700	20000	28.7	[27]
	Terrestrial	Human	/	70000	2000	10000	5.1	[28]
	Terrestrial	Horse	/	680000	2000	20000	9.8	[28]
	Terrestrial	Elephant	/	4000000	6000	6800	1.1	[29]
Insect (Terrestrial animals)	Terrestrial	Ant	/	0.00836	15	500	33.3	[30]
	Terrestrial	Spider	/	0.05	8.89	620	69.8	[31, 32]
	Terrestrial	American cockroach	/	0.83	30	1500	50	[33]
Aquatic animals	Aquatic	Tadpole	/	0.0091	28	500	17.9	[34]
	Aquatic	Water strider	/	0.01	11	1500	136.4	[35]
	Aquatic	Pike	/	20000	165	2100	12.7	[36]
	Aquatic	Bottlenose dolphin		300000	2610	6000	2.3	[37]
	Aquatic	Crocodile		1000000	1000	540	0.5	[37]
	Aquatic	Blue whale	/	100000000	26000	10000	0.4	[38]
Rigid robot	Terrestrial	DASH	Battery	16.2	100×100×50 (L×W×H)	1500	15	[39]
	Terrestrial	iSprawl	Battery	300	155×116×70 (L×W×H)	2300	15	[40]
	Terrestrial	Mini-Whegs	Battery	146	90×68×20 (L×W×H)	/	10	[41]

	Terrestrial	RHex	Battery	7500	530×200×150 (L×W×H)	550	1.04	[42]
	Terrestrial	MERbot II	Electroelastomer	130	140×160×90 (L×W×H)	135	0.857	[43]
	Terrestrial	Mobile robot	Piezo vibration	2.17	38×15×0.5 (L×W×H)	332	8.74	[44]
	Terrestrial	HAMR	Piezoelectric	1.27	43.7	442	10.1	[45]
	Terrestrial	Insect-scale robot	Piezoelectric	0.024	10	200	20	[46]
	Terrestrial	VelociRoACH	DC motor	30	100	2700	27	[47]
	Terrestrial	X2-VelociRoACH	DC motor	54	104	4900	47.1	[48]
Soft robot (Terrestrial)	Terrestrial	Tribot	Battery power	9.7	58×30×58 (L×W×H)	20	0.34	[49]
	Terrestrial	HAMR-F	Battery power	2.8	45	172	3.8	[50]
	Terrestrial	SEMR TST	Magnetic field	0.172	9	63	70	[51]
	Terrestrial	Quadrupedal microrobot	Magnetic field	0.001	2.5×1.6×0.7 (L×W×H)	37.3	14.9	[52]
	Terrestrial	Inchworm-inspired robot	Magnetic field	0.2	40	1.67	0.042	[53]
	Terrestrial	Limbless spinbot	Magnetic field	0.177	0.7	42	60	[54]
	Terrestrial	Hexapedal robot	Magnetic field	0.025	5×4×4 (L×W×H)	25.5	6.4	[55]
	Terrestrial	Caterpillar-inspired robot	Light exposure	/	8	0.25	0.031	[56]
	Terrestrial	LCE Caterpillar robot	Light exposure	~3.2	14.5	0.5	0.034	[57]
	Terrestrial	Inchworm-type	DE	0.2	20×10 (L×W)	21	1.03	[58]
	Terrestrial	Inchworm-mimic robot	DE	5	40	161	4	[59]

	Terrestrial	DEA	DE	0.97	40	12	0.3	60
	Terrestrial	Hopping-running robot	DE	6.5	85×48×500	518.3	6.10	61
	Terrestrial	DE bioinspired annelid robot	DE	10.3	170×120 (*L*×*W*)	5.3	0.031	62
	Terrestrial	Earthworm robot	DE	4.7	ϕ 20×45 (*D*×*L*)	2.5	0.0555	63
	Terrestrial	SJTU Roller Rolling robot	DE	0.88	49.66	36.27	0.73	64
	Terrestrial	Rolling rover	DE + Pneumatic	92	108	43	0.40	65
	Terrestrial	Multigait robot	Pneumatic	/	135.7×58.5×5.3 (*L*×*W*×*H*)	26.8	0.183	66
	Terrestrial / Aquatic	Amphibious climbing robot	Pneumatic	40	178.75	4.77	0.027	67
	Terrestrial	Mobile rolling robot	Pneumatic	210	80×63	5.74	0.07	68
	Terrestrial	LEAP runner	Pneumatics + DC motors	45	70×60 (*L*×*W*)	187.5	2.68	69
	Terrestrial	Hygrobot	Environmental humidity	0.035	25	6	0.24	70
	Terrestrial	Meshworm	Coiled NiTi fibrillar actuator	0.014	25	8	0.32	65
	Terrestrial	Softworm	SMA coils / motor tendons	/	200	112	0.56	71
	Terrestrial	Snakebot	Bidirectional fluidic elastomer	/	280×12×32 (*L*×*W*×*H*)	19	0.07	72
	Terrestrial	NUS crawler untethered robot	DE + electro-adhesion actuators	/	170	4.16	0.024	73
	Terrestrial	Ring-like robot	IPMC	/	57.3	8	0.14	74
	Terrestrial	Legged mobile robot	Bimorph PVDF actuator	0.32	50×10 (*L*×*W*)	35.3	0.706	75
	Terrestrial	Deformable robot	SMA	3.6	40	26	0.65	76

	Terrestrial/ Aquatic	Starfish robot	SMA	/	200	70	0.35	[77]
Soft robot (Aquatic)	Aquatic	Helical swimming micromachine	Magnetic field	/	0.035	0.32	9.1	[78]
		Wireless resonant magnetic microactuator	Magnetic field	0.000035	0.3×0.3×0.07 (L×W×H)	12.5	41.7	[79]
		swimming-sheet milli-robot	Magnetic field	0.0024	5.9	~100	17	[80]
		Electric fish	DE	90.3	93	64	0.69	[81]
		Underwater robotics	Cable-driven	333.5	160×95×80 (L×W×H)	40	0.25	[82]
		Swimming robot	Light exposure	0.0319	26.3	0.142	0.0054	[83]
		Half-spindle motor	Bubble release + Self-electrophoresis	/	0.1	7.5	75	[84]
		Turtle mimetic robot	SMA	588	290×260×70 (L×W×H)	11.5	0.0442	[85]
		Micro-robot fish	SMA	30	146×17×34 (L×W×H)	112	0.77	[86]
		IPMC walking robot	IPMCs	39	102×80×43 (L×W×H)	0.5	0.0048	[87]
		Biomimetic jellyfish robot	IPMCs	20	150×58 (D×H)	1.5	0.01	[88]
		Tadpole robot	IPMCs	16.2	96×24×25 (L×W×H)	23.6	0.25	[89]
		Water strider robot	Piezoelectric	1	100	30	0.3	[90]
		Micro-robot fish	Piezoelectric	1.93	60	45	0.75	[91]
		Resonant squid-inspired robot	DC Motor	380	266×59	~260	0.98	[92]

					(*L*×*D*)			
		Robotic fish	DC Motor	1300	440×316×80 (*L*×*W*×*H*)	530	1.21	93
		Tunabot	DC Motor	306	255×49×68 (*L*×*W*×*H*)	1000	4	94
		LEAP swimmer	Pneumatics + DC motors	51	150	117	0.78	32
	Aquatic (water-air interface swimmer)	Amphoteric polymer gels	Marangoni effect	0.1	7×4 (*D*×*H*)	50	7.143	95
		Pipet tip motor	Marangoni effect	/	30	90	3	8
		Camphor disk	Marangoni effect	/	3×1 (*D*×*H*)	60	20	9
		Benzoquinone disk	Marangoni effect	/	3×1 (*D*×*H*)	17.6 ± 4.1	5.87±1.37	10
		PDMS boat	Marangoni effect	1.41	26.4×12.7×6.4 (*L*×*W*×*H*)	85	3.22	12
		SU-8 boat	Marangoni effect	/	8.5×4.3×0.41 (*L*×*W*×*H*)	330	38.8	13
		SU-8 boat	Marangoni effect	/	24×6×1 (*L*×*W*×*H*)	140	5.83	14
		PNIPAm gel	Marangoni effect	3.58	7.4	310	41.89	15
		Polysulfone capsule	Marangoni effect	/	~2.46	157	63.8	16
		Poly(ADA-co-AA) gel	Marangoni effect	/	4×2 (*D*×*H*)	46.8	11.7	17
		Agarose gel	Marangoni effect	/	1×0.5 (*D*×*H*)	3	3	18
		Cellulose acetate	Marangoni effect	~0.01	14×0.055 (*D*×*H*)	150	10.71	19
		Multi-stage hydrogel rocket	Marangoni effect	/	9.4×5 (*L*×*W*)	16.2 ± 1.3	1.72	20
		Polyacrylamide hydrogel	Marangoni effect	/	1.3×5 (*D*×*H*)	2.2	0.44	21
		Peptide-MOF (HKUST-1)	Marangoni effect	/	1	31.8 ± 1.2	31.8	22

		PCN-222 MOF	Marangoni effect	0.0061	5×5×0.1	210	42	[23]
		Peptide-MOF (CuJAST-1)	Marangoni effect	2.34	1	67	67	[24]
		Chitosan hydrogel	Marangoni effect	0.025	2.5-10	178.2 to 265.8	71.28 to 106.32	[96]
		Camphor boats	Marangoni effect	0.02	3 (disk)<br>or 4 (rectangular)	~150	~50	[97]
		Alginate hydrogel	Marangoni effect	0.2-0.35	3	20 to 30	10 to 20	[98]
		Oil droplet	Marangoni effect	30 μL	41.0 ± 0.6	/	/	[99]
		Active particles	Marangoni effect	/	2 to 14	~20 to 100	5 to 10	[100]
		Our work 1	Marangoni effect	0.00001	0.31	894.28	2884.77	Present
		Our work 2	Marangoni effect	0.000014	0.36	956.29	2656.36	Present
		Our work 3	Marangoni effect	0.000068	0.64	669.35	1045.86	Present
		Our work 4	Marangoni effect	0.00023	1.00	517.27	517.27	Present
		Our work 5	Marangoni effect	0.00043	1.27	345.17	271.79	Present
		Our work 6	Marangoni effect	0.00312	2.67	307.68	115.24	Present
		Our work 7	Marangoni effect	0.01456	4.75	278.01	58.53	Present
		Our work 8	Marangoni effect	0.04095	7.00	258.57	36.94	Present
		Our work 9	Marangoni effect	0.1408	11.12	192.02	17.27	Present

D = diameter, *L* = length, *W* = width, *H* = height.

DE: Dielectric elastomer (DE); DC: Direct current (DC); IPMC: Ionic-polymer-metal composites (IPMC); SMA: Shape memory alloy (SMA);

Supplementary References

1 Davies, J. T. R., E. K. *Interfacial Phenomena*. (Academic: New York, 1963).

2 Kim, H., Muller, K., Shardt, O., Afkhami, S. & Stone, H. A. Solutal Marangoni flows of miscible liquids drive transport without surface contamination. *Nature Physics* **13**, 1105-1110 (2017). https://doi.org:10.1038/nphys4214

3 Bickel, T. & Detcheverry, F. Exact solutions for viscous Marangoni spreading. *Physical Review E* **106**, 045107 (2022).

4 Pena-Francesch, A., Giltinan, J. & Sitti, M. Multifunctional and biodegradable self-propelled protein motors. *Nature Communications* **10**, 3188 (2019). https://doi.org:10.1038/s41467-019-11141-9

5 Chen, Y.-J., Nagamine, Y. & Yoshikawa, K. Self-propelled motion of a droplet induced by Marangoni-driven spreading. *Physical Review E* **80**, 016303 (2009). https://doi.org:10.1103/PhysRevE.80.016303

6 Nagai, K., Sumino, Y., Kitahata, H. & Yoshikawa, K. Mode selection in the spontaneous motion of an alcohol droplet. *Physical Review E* **71**, 065301 (2005). https://doi.org:10.1103/PhysRevE.71.065301

7 Lidong Zhang, Y. Y., Xiaxin Qiu,Ting Zhang, Qing Chen, and Xinhua Huang. Marangoni Effect-Driven Motion of Miniature Robots and Generation of Electricity on Water. *Langmuir* **33**, 12609-12615 (2017). https://doi.org:10.1021/acs.langmuir.7b03270

8 Zhang, H., Duan, W., Liu, L. & Sen, A. Depolymerization-Powered Autonomous Motors Using Biocompatible Fuel. *Journal of the American Chemical Society* **135**, 15734-15737 (2013). https://doi.org:10.1021/ja4089549

9 Kitahata, H., Hiromatsu, S.-i., Doi, Y., Nakata, S. & Rafiqul Islam, M. Self-motion of a camphor disk coupled with convection. *Physical Chemistry Chemical Physics* **6**, 2409-2414 (2004). https://doi.org:10.1039/b315672a

10 Nobuhiko J. Suematsu, Y. M., Yui Matsuda, & Satoshi Nakata. Self-Motion of a benzoquinone disk coupled with a redox reaction. *Journal of Physical Chemistry C* **114**, 13340-13343 (2010). https://doi.org:10.1021/jp104666b

11 Keita Iida, N. J. S., Yumi Miyahara, Hiroyuki Kitahata, & Nakata, M. N. a. S. Experimental and theoretical studies on the self-motion of a phenanthroline disk coupled with complex formation. *Physical Chemistry Chemical Physics* **12**, 1557-1563 (2010). https://doi.org:10.1039/b918691c

12 Liu, X., Li, H., Qiao, L. & Luo, C. Driving mechanisms of CM-scaled PDMS boats of respective close and open reservoirs. *Microsystem Technologies* **17**, 875-889 (2011). https://doi.org:10.1007/s00542-010-1201-y

13 Luo, C., Qiao, L. & Li, H. Dramatic squat and trim phenomena of mm-scaled SU-8 boats induced by Marangoni effect. *Microfluidics and Nanofluidics* **9**, 573-577 (2010). https://doi.org:10.1007/s10404-010-0569-4

14 Qiao, L., Xiao, D., Lu, F. K. & Luo, C. Control of the radial motion of a self-propelled microboat through a side rudder. *Sensors and Actuators A: Physical* **188**, 359-366 (2012). https://doi.org:10.1016/j.sna.2012.04.004

15 Bassik, N., Abebe, B. T. & Gracias, D. H. Solvent Driven Motion of Lithographically Fabricated Gels. *Langmuir* **24**, 12158-12163 (2008). https://doi.org:10.1021/la801329g

16 Zhao, G., Seah, T. H. & Pumera, M. External-Energy-Independent Polymer Capsule Motors and Their Cooperative Behaviors. *Chemistry – A European Journal* **17**, 12020-12026 (2011). https://doi.org:10.1002/chem.201101450

17 J. P. Gong, S. M., M. Uchida, N. Isogai, and Y. Osada. Motion of Polymer Gels by Spreading Organic Fluid on Water. *Journal of Chemical Physics* **100**, 11092-11097 (1996).

https://doi.org:10.1021/jp960398e
18 Siowling Soh, K. J. M. B., and Bartosz A. Grzybowski. Dynamic Self-Assembly in Ensembles of Camphor Boats. *Journal of Physical Chemistry B* **112**, 10848-10853 (2008). https://doi.org:10.1021/jp7111457
19 Ioannis L. Liakos , P. S., Alice Scarpellini , Riccardo Carzino , Carlos Beltran ,Elisa Mele , Vittorio Murino , & Athanassia Athanassiou. Biomimetic Locomotion on Water of a Porous Natural Polymeric Composite. *Advanced Materials Interfaces* **3**, 1500854 (2016). https://doi.org:10.1002/admi.201500854
20 Yuling Liang, Y. X., Wei Ye, Dahua Yao, Yunhua Chen & Chaoyang Wang. Multi-stage hydrogel rockets with stage dropping-off by thermal/light stimulation. *Journal of Materials Chemistry A* **6**, 16838-16843 (2018). https://doi.org:10.1039/c8ta06715e
21 Sharma, R., Chang, S. T. & Velev, O. D. Gel-Based Self-Propelling Particles Get Programmed To Dance. *Langmuir* **28**, 10128-10135 (2012). https://doi.org:10.1021/la301437f
22 Yasuhiro Ikezoe, J. F., Tomasz L. Wasik, Menglu Shi, Takashi Uemura, & Susumu Kitagawa, H. M. Peptide–Metal Organic Framework Swimmers that Direct the Motion toward Chemical Targets. *Nano Letters* **15**, 4019-4023 (2015). https://doi.org:10.1021/acs.nanolett.5b00969
23 Park, J. H., Lach, S., Polev, K., Granick, S. & Grzybowski, B. A. Metal–Organic Framework “Swimmers” with Energy-Efficient Autonomous Motility. *ACS Nano* **11**, 10914-10923 (2017). https://doi.org:10.1021/acsnano.7b04644
24 Ikezoe, Y., Washino, G., Uemura, T., Kitagawa, S. & Matsui, H. Autonomous motors of a metal–organic framework powered by reorganization of self-assembled peptides at interfaces. *Nature Materials* **11**, 1081-1085 (2012). https://doi.org:10.1038/nmat3461
25 Anonymous. *5 Facts You May Not Know About Mice*, <https://www.moxieservices.com/blog/5-facts-you-may-not-know-about-mice/#:~:text=In%20a%20world%20where%20we,running%20up%20to%20160%20mph.> (2017).
26 O'Malley, C. I. *How Fast Can A Cat Run?*, <https://cats.com/how-fast-can-a-cat-run> (2017).
27 Benedict, M. *What Is The Fastest Dog Breed – 20 Fastest Dog Breeds*, <https://dogopedia.net/dog-facts/what-is-the-fastest-dog-breed-20-fastest-dog-breeds/> (2020).
28 Hildebrand, M. Motions of the Running Cheetah and Horse. *Journal of Mammalogy* **40**, 481-495 (1959). https://doi.org:10.2307/1376265
29 Hutchinson, J. R., Famini, D., Lair, R. & Kram, R. Are fast-moving elephants really running? *Nature* **422**, 493-494 (2003). https://doi.org:10.1038/422493a
30 Wittlinger, M., Wehner, R. d. & Wolf, H. The desert ant odometer: a stride integrator that accounts for stride length and walking speed. *Journal of Experimental Biology* **210**, 198-207 (2007). https://doi.org:10.1242/jeb.02657
31 Amaya, C. C., Klawinski, P. D. & Formanowicz, J. D. R. The Effects of Leg Autotomy on Running Speed and Foraging Ability in Two Species of Wolf Spider, (Lycosidae). *The American Midland Naturalist* **145**, 201-205 (2001). https://doi.org:10.1674/0003-0031(2001)145[0201:Teolao]2.0.Co;2
32 Resources. *brushlegged wolf spider*, <https://dnr.illinois.gov/content/dam/soi/en/web/dnr/education/cdindex/brushleggedwolfspider.pdf> (2020).
33 Robert J. Full, M. S. T. Mechanics of a rapid running insect two- four- and six-legged locomotion. *Journal of Experimental Biology* **156**, 215-231 (1991). https://doi.org:10.1242/jeb.156.1.215
34 Wilson, R., James, R. & Johnston, I. . Thermal acclimation of locomotor performance in tadpoles and adults of the aquatic frog Xenopus laevis. *Journal of Comparative Physiology B* **170**, 117-124 (2000).

https://doi.org:10.1007/s003600050266
35 Andersen, N. M. *A comparative study of loco motion on the water surface in semi aquatic bugs insecta hemiptera gerromorpha*. Vol. 139 337-396 (Videnskabelige Meddelelser fra Dansk Naturhistorisk Forening 1976).
36 Gray, J. The locomotion of fishes. *Essays in marine biology*, 1-16 (1953).
37 Elsworth, P. G., Seebacher, F. & Franklin, C. E. Sustained Swimming Performance in Crocodiles (Crocodylus porosus): Effects of Body Size and Temperature. *Journal of Herpetology* **37**, 363-368 (2003). https://doi.org:10.1670/0022-1511(2003)037[0363:Sspicc]2.0.Co;2
38 Kermack, K. A. The Propulsive Powers of Blue and Fin Whales. *Journal of Experimental Biology* **25**, 237-240 (1948). https://doi.org:10.1242/jeb.25.3.237
39 P. Birkmeyer, K. P. a. R. S. F. in *2009 IEEE/RSJ International Conference on Intelligent Robots and Systems* (IEEE, St. Louis, MO, USA, 2009).
40 Sangbae Kim, J. E. C., & Mark R. Cutkosky. iSprawl: Design and Tuning for High-speed Autonomous Open-loop Running. *International Journal of Robotics Research* **25** (2006). https://doi.org:10.1177/0278364906069150
41 J.M. Morrey, B. L., A.D. Horchler, R.E. Ritzmann, & R.D. Quinn. Highly mobile and robust small quadruped robots. *Proceedings 2003 IEEE/RSJ International Conference on Intelligent Robots and Systems (IROS 2003)* (2003). https://doi.org:10.1109/IROS.2003.1250609
42 Uluc Saranli, M. B., & Daniel E. Koditschek. RHex: A Simple and Highly Mobile Hexapod Robot. *The International Journal of Robotics Research* **20**, 616-631 (2001). https://doi.org:10.1177/02783640122067570
43 Qibing Pei, R. P., Marcus Rosenthal, Scott Stanford, Harsha Prahlad, & Roy Kornbluh in *Proceedings of SPIE* Vol. 5385 (San Diego, CA, United States, 2004).
44 Shin-ichi Aoshima, T. T., & Tetsuro Yabuta. A Miniature Mobile Robot Using Piezo Vibration for Mobility in a Thin Tube. *Journal of Dynamic Systems Measurement and Control* **115** (1993). https://doi.org:10.1115/1.2899031
45 Baisch, A. T., Ozcan, O., Goldberg, B., Ithier, D. & Wood, R. J. High speed locomotion for a quadrupedal microrobot. *The International Journal of Robotics Research* **33**, 1063-1082 (2014). https://doi.org:10.1177/0278364914521473
46 Yichuan Wu, J. K. Y., Jiaming Liang, Zhichun Shao, Mingjing Qi, Junwen Zhong, Zihao Luo, Xiaojun Yan, Min Zhang, Xiaohao Wang, Ronald S. Fearing, Robert J. Full, & Liwei Lin. Insect-scale fast moving and ultrarobust soft robot. *SCIENCE ROBOTICS* **4** (2019). https://doi.org:10.1126/scirobotics.aax1594
47 Duncan W. Haldane, K. C. P., Fernando L. Garcia Bermudez, & Ronald S. Fearing. Animal-inspired design and aerodynamic stabilization of a hexapedal millirobot. *2013 IEEE International Conference on Robotics and Automation (ICRA)* (2013). https://doi.org:10.1109/ICRA.2013.6631034
48 Duncan W. Haldane, R. S. F. Running beyond the bio-inspired regime. *2015 IEEE International Conference on Robotics and Automation (ICRA)* (2015). https://doi.org:10.1109/ICRA.2015.7139828
49 Zhakypov, Z., Mori, K., Hosoda, K. & Paik, J. Designing minimal and scalable insect-inspired multi-locomotion millirobots. *Nature* **571**, 381-386 (2019). https://doi.org:10.1038/s41586-019-1388-8
50 Benjamin Goldberg , R. Z., Neel Doshi , Elizabeth Farrell Helbling , Griffin Whittredge, Mirko Kovac, & Robert J. Wood. Power and Control Autonomy for High-Speed Locomotion With an Insect-Scale Legged Robot. *IEEE Robotics and Automation Letters* **3**, 987-993 (2018). https://doi.org:10.1109/lra.2018.2793355
51 Guoyong Mao , D. S., Doris Danninger,, Bekele Hailegnaw, F. H., Thomas Stockinger, MichaelDrack,

& Nikita Arnold, M. K. Ultrafast small-scale soft electromagnetic robots. *Nature Communications* **13**, 4456 (2022). https://doi.org:10.1038/s41467-022-32123-4
52 Ryan St. Pierre, W. G., & Sarah Bergbreiter. A 3d-Printed 1 Mg Legged Microrobot Running at 15 Body Lengths Per Second. *2018 Solid-State, Actuators, and Microsystems Workshop* **3** (2018). https://doi.org:10.31438/trf.hh2018.16
53 Joyee, E. B. & Pan, Y. A Fully Three-Dimensional Printed Inchworm-Inspired Soft Robot with Magnetic Actuation. *Soft Robotics* **6**, 333-345 (2019). https://doi.org:10.1089/soro.2018.0082
54 Won, S., Kim, S., Park, J. E., Jeon, J. & Wie, J. J. On-demand orbital maneuver of multiple soft robots via hierarchical magnetomotility. *Nature Communications* **10**, 4751 (2019). https://doi.org:10.1038/s41467-019-12679-4
55 Dana Vogtmann, R. S. P., & Sarah Bergbreiter. A 25 MG magnetically actuated microrobot walking at > 5 body lengths/sec. *2017 IEEE 30th International Conference on Micro Electro Mechanical Systems (MEMS)* (2017). https://doi.org:10.1109/MEMSYS.2017.7863370
56 Hao Zeng, O. M. W., Piotr Wasylczyk, & Arri Priimagi. Light-Driven, Caterpillar-Inspired Miniature Inching Robot. *Macromolecular Rapid Communications* **39**, 1700224 (2017). https://doi.org:10.1002/marc.201700224
57 Rogóż, M., Zeng, H., Xuan, C., Wiersma, D. S. & Wasylczyk, P. Light-Driven Soft Robot Mimics Caterpillar Locomotion in Natural Scale. *Advanced Optical Materials* **4**, 1689-1694 (2016). https://doi.org:10.1002/adom.201600503
58 Mihai Duduta, D. R. C., & Robert J. Wood. A high speed soft robot based on dielectric elastomer actuators. *Mihai Duduta; David R. Clarke; Robert J. Wood* (2017). https://doi.org:10.1109/ICRA.2017.7989501
59 Tiefeng Li, Z. Z., Guoyong Mao, Xuxu Yang, Yiming Liang, Chi Li, Shaoxing Qu, Zhigang Suo, & Wei Yang. Agile and Resilient Insect-Scale Robot. *Soft Robotics* **6**, 133-141 (2019). https://doi.org:10.1089/soro.2018.0053
60 Xiaobin Ji, X. L., Vito Cacucciolo, Matthias Imboden, Yoan Civet, Alae El Haitami, Sophie Cantin, Yves Perriard, & Herbert Shea. An autonomous untethered fast soft robotic insect driven by low-voltage dielectric elastomer actuators. *SCIENCE ROBOTICS* **4** (2019). https://doi.org:10.1126/scirobotics.aaz6451
61 Jianwen Zhao, J. Z., David McCoul, Zhaogang Hao, Shu Wang, Xinbo Wang, Bo Huang, & Lining Sun. Soft and Fast Hopping–Running Robot with Speed of Six Times Its Body Length Per Second. *Soft Robotics* **6**, 713-721 (2019). https://doi.org:10.1089/soro.2018.0098
62 Liang Xu, H.-Q. C., Jiang Zou, Wan-Ting Dong, Guo-Ying Gu, Li-Min Zhu, & Xiang-Yang Zhu. Bio-inspired annelid robot: a dielectric elastomer actuated soft robot. *Bioinspiration & Biomimetics* **12**, 025003 (2017). https://doi.org:10.1088/1748-3190/aa50a5
63 Jung, K., Koo, J. C., Nam, J.-d., Lee, Y. K. & Choi, H. R. Artificial annelid robot driven by soft actuators. *Bioinspiration & Biomimetics* **2**, S42-S49 (2007). https://doi.org:10.1088/1748-3182/2/2/s05
64 Li, W.-B., Zhang, W.-M., Zou, H.-X., Peng, Z.-K. & Meng, G. A Fast Rolling Soft Robot Driven by Dielectric Elastomer. *IEEE/ASME Transactions on Mechatronics* **23**, 1630-1640 (2018). https://doi.org:10.1109/tmech.2018.2840688
65 Sangok Seok, C. D. O., Kyu-Jin Cho, Robert J. Wood, Daniela Rus, & Sangbae Kim. Meshworm: A Peristaltic Soft Robot With Antagonistic Nickel Titanium Coil Actuators. *IEEE/ASME Transactions on Mechatronics* **18**, 1485-1497 (2013). https://doi.org:10.1109/tmech.2012.2204070
66 Robert F. Shepherd, F. I., Wonjae Choi, Stephen A. Morin, Adam A. Stokes, Aaron D. Mazzeo, Xin Chen,

& Michael Wang, G. M. W. Multigait soft robot. *Proceedings of the National Academy of Sciences* **108**, 20400-20403 (2011). https://doi.org:10.1073/pnas.1116564108
67 Yichao Tang, Q. Z., Gaojian Lin, & Jie Yin. Switchable Adhesion Actuator for Amphibious Climbing Soft Robot. *Soft Robotics* **5**, 592-600 (2018). https://doi.org:10.1089/soro.2017.0133
68 Cagdas D. Onal, X. C., George M. Whitesides, & Daniela Rus. Soft Mobile Robots with On-Board Chemical Pressure Generation. *Robotics Research*, 525-540 (2017). https://doi.org:10.1007/978-3-319-29363-9_30
69 Yichao Tang, Y. C., Jiefeng Sun, Tzu-Hao Huang, Omid H. Maghsoudi, & Andrew Spence, J. Z., Hao Su, & Jie Yin. Leveraging elastic instabilities for amplified performance: Spine-inspired high-speed and high-force soft robots. *SCIENCE ADVANCES* **6**, eaaz6912 (2020). https://doi.org:10.1126/sciadv.aaz6912
70 Beomjune Shin, J. H., Minhee Lee, Keunhwan Park, Gee Ho Park, Tae Hyun Choi, & Kyu-Jin Cho, H.-Y. K. Hygrobot: A self-locomotive ratcheted actuator powered by environmental humidity. *SCIENCE ROBOTICS* **3**, eaar2629 (2018). https://doi.org:10.1126/scirobotics.aar2629
71 T Umedachi, V., & BATrimmer. Softworms: the design and control of non-pneumatic, 3D-printed, deformable robots. *Bioinspiration & Biomimetics* **11**, 025001 (2016). https://doi.org:10.1088/1748-3190/11/2/025001
72 Cagdas D Onal, D. R. Autonomous undulatory serpentine locomotion utilizing body dynamics of a fluidic soft robot. *Bioinspiration & Biomimetics* **8**, 026003 (2013). https://doi.org:10.1088/1748-3182/8/2/026003
73 Jiawei Cao, L. Q., Jun Liu, Qinyuan Ren, Choon Chiang Foo, Hongqiang Wang, & Heow Pueh Lee, J. Z. Untethered soft robot capable of stable locomotion using soft electrostatic actuators. *Extreme Mechanics Letters* **21**, 9-16 (2018). https://doi.org:10.1016/j.eml.2018.02.004
74 A Firouzeh, M. O., A Alasty, & A Iraji zad. An IPMC-made deformable-ring-like robot. *Smart Materials and Structures* **21**, 065011 (2012). https://doi.org:10.1088/0964-1726/21/6/065011
75 Tongil Park, Y. C. Soft mobile robot inspired by animal-like running motion. *Scientific Reports* **9**, 14700 (2019). https://doi.org:10.1038/s41598-019-51308-4
76 Sugiyama, Y. & Hirai, S. Crawling and Jumping by a Deformable Robot. *The International Journal of Robotics Research* **25**, 603-620 (2016). https://doi.org:10.1177/0278364906065386
77 HuJin, E. D., Gursel Alici, Shixin Mao, Xu Min, Chunshan Liu, KHLow, & Jie Yang. A starfish robot based on soft and smart modular structure (SMS) actuated by SMA wires. *Bioinspiration & Biomimetics* **11**, 056012 (2016). https://doi.org:10.1088/1748-3190/11/5/056012
78 Soichiro Tottori, L. Z., Famin Qiu, Krzysztof K. Krawczyk, Alfredo Franco-Obregón, & Bradley J. Nelson. Magnetic Helical Micromachines: Fabrication, Controlled Swimming, and Cargo Transport. *Advanced Materials* **24**, 811-816 (2012). https://doi.org:10.1002/adma.201103818
79 Karl Vollmers, D. R. F., Bradley E. Kratochvil, & Bradley J. Nelson. Wireless resonant magnetic microactuator for untethered mobile. *Applied Physics Letters* **92**, 144103 (2008). https://doi.org:10.1063/1.2907697
80 Eric Diller, J. Z., Guo Zhan Lum, Matthew R. Edwards, & Metin Sitti. Continuously distributed magnetization profile for millimeter-scale elastomeric undulatory swimming. *Applied Physics Letters* **104**, 174101 (2014). https://doi.org:10.1063/1.4874306
81 Tiefeng Li, G. L., Yiming Liang, Tingyu Cheng, Jing Dai, Xuxu Yang, Bangyuan Liu, Zedong Zeng, Zhilong Huang, Yingwu Luo, Tao Xie, & Wei Yang. Fast-moving soft electronic fish. *SCIENCE ADVANCES* **3**, e1602045 (2017). https://doi.org:10.1126/sciadv.1602045

82 Giorgio Serchi, F., Arienti, A. & Laschi, C. Biomimetic Vortex Propulsion: Toward the New Paradigm of Soft Unmanned Underwater Vehicles. *IEEE/ASME Transactions on Mechatronics* **18**, 484-493 (2013). https://doi.org:10.1109/tmech.2012.2220978

83 Chaolei Huang, J.-a. L., Xiaojun Tian, Yuechao Wang, Yanlei Yu, & Jie Liu. Miniaturized Swimming Soft Robot with Complex Movement Actuated and Controlled by Remote Light Signals. *Scientific Reports* **5**, 17414 (2015). https://doi.org:10.1038/srep17414

84 Zhou, C., Zhu, P., Tian, Y., Xu, M. & Wang, L. Engineering Micromotors with Droplet Microfluidics. *ACS Nano* **13**, 6319-6329 (2019). https://doi.org:10.1021/acsnano.9b00731

85 Sung-Hyuk Song, M.-S. K., Hugo Rodrigue, Jang-Yeob Lee, Jae-Eul Shim, Min-Cheol Kim, Won-Shik Chu, & Sung-Hoon Ahn. Turtle mimetic soft robot with two swimming gaits. *Bioinspiration & Biomimetics* **11**, 036010 (2016). https://doi.org:10.1088/1748-3190/11/3/036010

86 Wang, Z., Hang, G., Li, J., Wang, Y. & Xiao, K. A micro-robot fish with embedded SMA wire actuated flexible biomimetic fin. *Sensors and Actuators A: Physical* **144**, 354-360 (2008). https://doi.org:10.1016/j.sna.2008.02.013

87 Chang, Y.-c. & Kim, W.-j. Aquatic Ionic-Polymer-Metal-Composite Insectile Robot With Multi-DOF Legs. *IEEE/ASME Transactions on Mechatronics* **18**, 547-555 (2013). https://doi.org:10.1109/tmech.2012.2210904

88 Joseph Najem, S. A. S., Barbar Akle, & Donald J Leo. Biomimetic jellyfish-inspired underwater vehicle actuated by ionic polymer metal composite actuators. *Smart Materials and Structures* **21**, 094026 (2012). https://doi.org:10.1088/0964-1726/21/9/094026

89 Kim, B., Kim, D.-H., Jung, J. & Park, J.-O. A biomimetic undulatory tadpole robot using ionic polymer–metal composite actuators. *Smart Materials and Structures* **14**, 1579-1585 (2005). https://doi.org:10.1088/0964-1726/14/6/051

90 Yun Seong, S. & Sitti, M. Surface-Tension-Driven Biologically Inspired Water Strider Robots: Theory and Experiments. *IEEE Transactions on Robotics* **23**, 578-589 (2007). https://doi.org:10.1109/tro.2007.895075

91 Quanliang Zhao, S. L., Jinghao Chen, Guangping He, Jiejian Di, Lei Zhao,Tingting Su, Mengying Zhang, & Zhiling Hou. Fast-moving piezoelectric micro-robotic fish with double caudal fins. *Robotics and Autonomous Systems* **140**, 103733 (2021). https://doi.org:10.1016/j.robot.2021.103733

92 Thierry Bujard, F. G.-S., & Gabriel D. Weymouth. A resonant squid-inspired robot unlocks biological propulsive efficiency. *SCIENCE ROBOTICS* **6**, eabd2971 (2021). https://doi.org:10.1126/scirobotics.abd2971

93 Zhang, S., Qian, Y., Liao, P., Qin, F. & Yang, J. Design and Control of an Agile Robotic Fish With Integrative Biomimetic Mechanisms. *IEEE/ASME Transactions on Mechatronics* **21**, 1846-1857 (2016). https://doi.org:10.1109/tmech.2016.2555703

94 J. Zhu, C. W., D. K. Wainwright, V. Di Santo, G. V. Lauder, & H. Bart-Smith. Tuna robotics: A high-frequency experimental platform exploring the performance space of swimming fishes. *SCIENCE ROBOTICS* **4**, eaax4615 (2019). https://doi.org:10.1126/scirobotics.aax4615

95 Yoshihito Osada, J. G., Makoto Uchida, & Noriharu Isogai Noriharu Isogai. Spontaneous Motion of Amphoteric Polymer Gels on Water. *Japanese Journal of Applied Physics* **34**, L511-L512 (1995). https://doi.org:10.1143/JJAP.34.L511

96 Kumar, P., Horváth, D. & Tóth, Á. Sol-gel transition programmed self-propulsion of chitosan hydrogel. *Chaos: An Interdisciplinary Journal of Nonlinear Science* **32** (2022). https://doi.org/10.1063/5.0097035

97 Suematsu, N. J., Sasaki, T., Nakata, S. & Kitahata, H. Quantitative estimation of the parameters for self-motion driven by difference in surface tension. *Langmuir* **30**, 8101-8108 (2014). https://doi.org/10.1021/la501628d

98 Ender, H., Froin, A.-K., Rehage, H. & Kierfeld, J. Surfactant-loaded capsules as Marangoni microswimmers at the air–water interface: Symmetry breaking and spontaneous propulsion by surfactant diffusion and advection. *The European Physical Journal E* **44**, 21 (2021). https://doi.org/10.1140/epje/s10189-021-00035-8

99 Tanaka, S., Nakata, S. & Nagayama, M. A surfactant reaction model for the reciprocating motion of a self-propelled droplet. *Soft Matter* **17**, 388-396 (2021). https://doi.org/10.1039/d0sm01500h

100 Boniface, D., Cottin-Bizonne, C., Kervil, R., Ybert, C. & Detcheverry, F. Self-propulsion of symmetric chemically active particles: Point-source model and experiments on camphor disks. *Physical Review E* **99**, 062605 (2019). https://doi.org/10.1103/PhysRevE.99.062605
