# Supplementary material for: Sustained interfacial powering through self-generated mantle and siphon of a gelling droplet
Source: Nat Commun. 2026 Feb 10;17:2566. doi: 10.1038/s41467-026-69481-2 (PMC13000242; doi:10.1038/s41467-026-69481-2)
Supplement: Supplementary file 2 — Description of Additional Supplementary Files [file 41467_2026_69481_MOESM2_ESM.pdf]

File Name: Supplementary Movie 1

Description: Soap-boat-propulsion comparison.

File Name: Supplementary Movie 2

Description: The long-lifetime self-propulsion of the gelling droplet.

File Name: Supplementary Movie 3

Description: Spreading and gelation of sodium alginate droplet without and with surfactants.

File Name: Supplementary Movie 4

Description: Visualization of anisotropic surfactant release.

File Name: Supplementary Movie 5

Description: Schlieren visualization of pulsed motion.

File Name: Supplementary Movie 6

Description: Impact of continuous crosslinking.

File Name: Supplementary Movie 7

Description: Numerically-calculated motor propulsion and surfactant concentration distribution.

File Name: Supplementary Movie 8

Description: Powering the wireless sensing system.

File Name: Supplementary Movie 9

Description: Powering and controlling of the gear mechanism.

File Name: Supplementary Movie 10

Description: Powering and controlling of free interfacial machines.

File Name: Supplementary Movie 11

Description: Powering pinned interfacial machines.

File Name: Supplementary Movie 12

Description: Powering the translational and swinging cam mechanism.

File Name: Supplementary Movie 13

Description: Powering the crank-rocker mechanism.

File Name: Supplementary Movie 14

Description: Powering a reciprocating slider.
